# Arabinan saccharification by biogas reactor metagenome-derived arabinosyl hydrolases

**DOI:** 10.1186/s13068-022-02216-9

**Published:** 2022-11-12

**Authors:** Yajing Liu, Angel Angelov, Werner Feiler, Melanie Baudrexl, Vladimir Zverlov, Wolfgang Liebl, Sonja Vanderhaeghen

**Affiliations:** 1 Chair of Microbiology, Technical University of Munich, Emil-Ramann-Straβe 4, 85354 Freising-Weihenstephan, Germany; 2 Present Address: Chair of Chemistry of Biogenic Resources, Technical University of Munich, Schulgasse 16, 94315 Straubing, Germany; 3 Present Address: NGS Competence Center Tübingen, Universitätsklinikum Tübingen, Calwerstraße 7, 72076 Tübingen, Germany; 4 Present Address: IMGM Laboratories, Lochhamer Straße 29a, 82152 Planegg, Germany

**Keywords:** Metagenomics, Sugar beet pulp degradation, *Xylanivirga thermophila*, Arabinosyl hydrolases, Pentose metabolism

## Abstract

**Background:**

Plant cell walls represent the most plentiful renewable organic resource on earth, but due to their heterogeneity, complex structure and partial recalcitrance, their use as biotechnological feedstock is still limited.

**Results:**

In order to identify efficient enzymes for polysaccharide breakdown, we have carried out functional screening of metagenomic fosmid libraries from biogas fermenter microbial communities grown on sugar beet pulp, an arabinan-rich agricultural residue, or other sources containing microbes that efficiently depolymerize polysaccharides, using CPH (chromogenic polysaccharide hydrogel) or ICB (insoluble chromogenic biomass) labeled polysaccharide substrates. Seventy-one depolymerase-encoding genes were identified from 55 active fosmid clones by using Illumina and Sanger sequencing and dbCAN CAZyme (carbohydrate-active enzyme) annotation. An around 56 kb assembled DNA fragment putatively originating from *Xylanivirga thermophila* strain or a close relative was analyzed in detail. It contained 48 ORFs (open reading frames), of which 31 were assigned to sugar metabolism. Interestingly, a large number of genes for enzymes putatively involved in degradation and utilization of arabinose-containing carbohydrates were found. Seven putative arabinosyl hydrolases from this DNA fragment belonging to glycoside hydrolase (GH) families GH51 and GH43 were biochemically characterized, revealing two with endo-arabinanase activity and four with exo-α-l-arabinofuranosidase activity but with complementary cleavage properties. These enzymes were found to act synergistically and can completely hydrolyze SBA (sugar beet arabinan) and DA (debranched arabinan).

**Conclusions:**

We screened 32,776 fosmid clones from several metagenomic libraries with chromogenic lignocellulosic substrates for functional enzymes to advance the understanding about the saccharification of recalcitrant lignocellulose. Seven putative *X. thermophila* arabinosyl hydrolases were characterized for pectic substrate degradation*.* The arabinosyl hydrolases displayed maximum activity and significant long-term stability around 50 °C. The enzyme cocktails composed in this study fully degraded the arabinan substrates and thus could serve for arabinose production in food and biofuel industries.

**Supplementary Information:**

The online version contains supplementary material available at 10.1186/s13068-022-02216-9.

## Background

A huge amount of agricultural waste is produced worldwide every year, which represents an economically important source of sugars for biotechnological production and reduce the need for petroleum-based resources [1]. Sugar beet pulp (SBP) is a biodegradable waste product of sugar production generated in massive amounts, with around 17 million tons of sugar beet residues generated each year worldwide [2, 3]. SBP consists mainly of 22–30% cellulose, 24–32% of hemicelluloses, 24–32% pectin and 3–4% of lignin [3]. Each component has its inherent complexity or heterogeneity. Therefore, SBP bioconversion into more simple chemical compounds or polymers remains a great challenge for industrial degradation [4]. It can be exemplified by pectin, a structural heteropolysaccharide with a molecular weight of 60,000–130,000 g mol^−1^ [5], which contains high amounts of galacturonic acid and arabinose (each 1/5 to 1/4 of pulp dry weight) [6]. The backbone of pectin is usually composed of α-1,4-linked galacturonic acid residues (homogalacturonan) or alternatively of repeating dimers of α-d-galacturonic acid and α-l-1,2-linked rhamnose (rhamnogalacturonan I) [7]. Rhamnogalacturonan I (RGI) can be highly substituted with various sugar branches, mainly consisting of α-1,5-linked arabinan with side chains attached to the O-2 or O-3 position of arabinose, as well as 1,4-linked β-galactans with a low degree of polymerization and highly branched 1,3/6-linked galactans [7]. The formation of a “hairy” structure makes the compound sterically inaccessible and in consequence difficult to be degraded by pectinolytic enzymes.

In nature, microorganisms have evolved sophisticated strategies for the degradation of plant biomass, such as the adoption of multienzyme cellulosome systems in certain anaerobes or the formation of complex cell surface-bound structures encoded in polysaccharide utilization loci (PULs) [8, 9], with up to 50 PULs for a range of substrates in some bacteria [10]. Mono- and oligosaccharides released can serve as carbon and energy sources for their growth. Carbohydrate-active enzymes, some of which contain carbohydrate-binding modules (CBMs), can be broadly classified into five different groups, including polysaccharide lyases, glycoside hydrolases (GHs), carbohydrate esterases, glycosyltransferases, plus a range of auxiliary enzymes, based on their activity and specific structure features [11]. Usually, the enzymatic synergism of different carbohydrate-active enzymes (CAZymes) plays an important role in deconstructing plant cell wall biomass [12]. One example is the hydrolysis of arabinans which requires the synergistic action of different arabinosyl hydrolases [13]. Endo-α-l-1,5-arabinanases which are so far only found in family GH43 randomly cleave the arabinan backbone. Exo-α-l-arabinofuranosidases, which are encountered in the families GH2, GH3, GH5, GH39, GH43, GH51, GH54, GH62 and are active on 1,2-,1,3-arabinosyl side-chain substitutions or terminal 1,5-linked backbone residues [11, 13]. Besides, exo-α-l-1,5-arabinanase activity has been described mainly in family GH93 and also in some representatives from family GH43 which releases arabinose, arabinobiose or arabinotriose as a major products from linear arabinan [14–17].

Diverse repertoires of polysaccharide-degrading enzymes can be found in various bacteria, including *Bacteroidetes*, *Firmicutes*, *Spirochaetes* [18]. Bacteria and their enzymes isolated from different natural or anthropogenic environments are usually well adapted to their surroundings by virtue of specific metabolic characteristics and substrate specificity features, respectively. For example, microbes in biogas fermenters are naturally specialized in saccharification of lignocellulolytic biomass. Thus, they are ideal targets for research aiming to unveil functions of lignocellulosic plant biomass degradation [19].

To investigate the repertoire of enzymes used by microbes for the deconstruction of plant biomass, a standard approach is to isolate bacterial or fungal strains that have a naturally strong lignocellulosic degradation ability, followed by whole-genome sequencing of single strains to identify genes and enzymes of interest [20, 21]. But so far, merely a small portion of the microbes from complex communities have been cultivated as pure cultures. Therefore, many bacteria are underrepresented when using culture-dependent method, limiting our knowledge about the functional and ecological traits of biomass decomposer populations [22]. This bottleneck issue has been partially overcome by the development of metagenomics, where the genetic material is directly extracted from the environment. Thereafter, enzyme-encoding genes are found either via the function-based screening of metagenomic libraries and/or via sequence-driven analyses [23]. Through function-based screening, enzymes with desired catalytic activities can be obtained without any prior information about their sequences, so this is an efficient method to identify novel enzyme families or to identify within known enzyme families new enzymes with functions previously unseen in those families [24]. Also, function-based metagenomic screening contributes to rationalizing sequencing efforts by focusing specifically on enzymes of biotechnological interest [25].

In the present study, we constructed metagenomic libraries with DNA isolated from SBP-fed biogas fermenter microbial communities, using *E. coli* as host. These libraries and other existing libraries from anaerobic plant-biomass degrading communities were subjected to function-based screening using in-house CPH substrates (CPH-arabinan, -arabinoxylan, -xylan, -pectin, -CMC) and an ICB substrate (ICB-SBP). After sequence analysis and identification of genes for putative degradative enzymes, our goal in this study was to focus on a number of enzymes of decomposition of arabinose-containing polysaccharides which were all encoded on a large assembled metagenomic DNA fragment. This fragment apparently originated from a bacterium closely related to *Xylanivirga thermophila,* a *Clostridiales* member of the phylum *Bacillota (*syn. *Firmicutes*). We hypothesized that an accumulation of numerous putative arabinosyl hydrolase genes close together on a sequence contig may indicate high potential for degradation of arabinose-containing polysaccharides by these enzymes, which also may be useful for biotechnological application.

## Results

### Activity-based metagenomic library screening

A function-based screening of 32,776 fosmid clones was performed to identify enzyme activities with application potential for sugar beet pulp (SBP) saccharification, including xylanase, arabinoxylanase, arabinanase, CMCase and pectinase activity, as well as SBP-degrading activity. 25,000 of these fosmid clones were constructed from metagenomic DNA isolated from microbial communities which originated from thermophilic biogas fermenters and were propagated anaerobically in a SBP-containing medium (Table 1). Based on 16S rRNA amplicon sequencing, this SBP-utilizer-enriched fermenter sample included *Firmicutes* (88.66% in MOD18 and 82.12% in T1T2), *Thermotogae* (6.28% in MOD18 and 17.22% in T1T2) and *Bacteroidetes* (4.96% in MOD18 and 0.03% in T1T2) as the major phyla (Table S1). The remaining 7,776 fosmid clones were constructed from different environment samples, among them were clones from an elephant feces metagenomic library available in our lab. The rationale for this lies in the fact that elephants are herbivorous animals and therefore plant cell wall-degrading microorganisms can be expected in fecal samples. Similarly, other already available fosmid clones constructed from samples putatively containing plant cell wall-degrading organisms were included in the screening. This effort resulted in a total of 55 unique, positive fosmid clones with various activities which were subjected to sequencing (Table 1), including 31 positive fosmid clones with CMCase activity, 6 with xylanase activity, 6 with arabinanase activity, 3 with arabinoxylanase activity and 9 with dual enzyme activity (Table 1, Additional file 1: Fig. S1). Among those positive fosmid clones, 40 had pCC1FOS and 15 had pCT3FK as the backbone.

**Table 1:** Construction and functional screening of metagenomic libraries to identify degradative enzymes

Name of libraries	Fosmid backbones	Origins	Number of clones	Size of inserts	Positive clones for sequencing
ED library	pCT3FK	Elephant feces	1372	~ 40 kb	3 (2 CMCases^a^, 1 xylanase)
BM1T library	pCT3FK	Thermophilic digestate, fermenter	980	~ 40 kb	15 (3 arabinoxylanases, 9 dual activities^b^, 3 xylanases)
MOD18 library	pCC1FOS	Thermophilic digestate, fermenter, 50 °C	20000	~ 40 kb	18 (4 arabinanases, 14 CMCases)
T1T2 library	pCC1FOS	Thermophilic digestate, fermenter, 55 °C	5000	~ 40 kb	19 (2 arabinanases, 2 xylanases, 15 CMCases)
Others	pCC1FOS/pCT3FK	various	5424	~ 40 kb	0
Total			32776		55

Positive clones were screened by using chromogenic polysaccharide hydrogel (CPH) substrates and insoluble chromogenic biomass (ICB) substrates

^a^CMCases: carboxymethyl cellulases

^b^Dual activities: both xylanase and arabinoxylanase activities

### Sequence analysis by “pooled sequencing”

Sequence information from the fosmids conferring glycoside hydrolase activity was obtained with a combination of Illumina and Sanger sequencing (named “pooled sequencing” strategy). Instead of using barcodes to track sequence reads from individual fosmid clones, the fosmid inserts were sequenced at their termini by Sanger end sequencing to assign the fosmids to the contigs generated from Illumina shotgun sequencing of pools of fosmid DNAs.

Of the 55 sequenced fosmid clones, 4 clones repeatedly failed Sanger end sequencing and were excluded from subsequent analyses. The remaining 102 end sequencing results were used for further analysis. After shotgun sequencing, 258 contigs were obtained (130 contigs from the pCC1FOS fosmid pool and 128 from the pCT3FK fosmid pool), which included 54 contigs encoding putative CAZymes from the biogas fermenter communities enriched on SBP and 70 contigs encoding putative CAZymes from either the thermophilic digestate or the elephant feces sample (Additional file 1Table S2), whereby putative CAZymes were annotated by the dbCAN database. The average lengths of the contigs from the pCC1FOS and the pCT3FK fosmid pools were about 37 kb (Additional file 1: Table S2). Contigs far exceeding this length were found to be *E. coli* genomic DNA contaminations and were excluded from subsequent analyses. Of the remaining 81 non-*E. coli* contigs, which have an average length of about 8 kb, 39 were from the pCC1FOS fosmid pool and 42 from the pCT3FK fosmid pool. 54 of the 81 contigs could be assigned to fosmid clones with known function by Sanger end sequencing, and about 57% of these contigs encoded more than one putative CAZyme (Table 2). 47% of the recombinant fosmids included in sequencing had a sequence gap in the coverage in either the central or terminal region of the insert DNA.

**Table 2:** Summary of “non-*E. coli*” contigs from shotgun sequencing of positive fosmid clones

Retrieved non-*E. coli* contigs^a^	pCC1FOS fosmid pool (40 clones)	pCT3FK fosmid pool (15 clones)	Both pools
Number of contigs	39	42	81
Number of retrieved contigs^b^	32	22	54
No. of contigs with CAZymes^c^	13	18	31
Total length of contigs (bps)	295,166	344,179	639,345
Average length of contigs (bps)^d^	7568	8194	7893

Positive clones were screened by using chromogenic polysaccharide hydrogel (CPH) substrates and insoluble chromogenic biomass (ICB) substrates

^a^Contigs from *E. coli* genomic DNA contamination or from vector backbone were confirmed by the size of contig (far more than 40 kb) and sequence identity searched against NCBI database

^b^Retrieved contigs refer to the contigs which could be aligned to at least one Sanger end sequence

^c^CAZymes were predicted by dbCAN database

^d^Average length of contigs was calculated by using the sum of the contig lengths divided by the amount of contigs after filtering out the *E. coli* originated contigs

By searching against the dbCAN database, putative glycoside hydrolase (GH) genes encoding 71 putative CAZymes from 26 different GH families were identified within the metagenome dataset (Table 3), including members from GH43, GH10, GH51, GH2 and GH11. Representatives of GH43 and GH10 were the most frequent putative GHs found in the microbiome of the environmental samples (Table 3). Members of GH43 and GH10 are involved in the breakdown of hemicellulose and pectic substrates. In comparison to the large numbers of CMCase-positive clones (31 out of in total 55 clones) identified by functional screening, there were only a few unique genes (in total 9 genes) identified on the contigs, which encode putative enzymes related to cellulose/CMC hydrolysis (Table 3). Surprisingly, no putative pectinase-encoding genes were found, neither from the functional library screening nor by sequence analysis of the generated contigs, even though the microbial communities from the fermenter samples efficiently degraded the pectin-rich biomass SBP. On the other hand, some genes were identified on clone inserts which due to their functional annotation could be involved in the degradation of pectin side chains, such as arabinan and galactan in the side chain of RG I.

**Table 3:** Statistics of genes for putative fibrolytic enzymes from 55 sequenced fosmids

CAZy family^a^	Putative activity ^b^	Gene no.
Hemicellulose degrading ability (37 putative CAZymes)		
GH10	Endo-1,4-beta-xylanase [EC:3.2.1.8]	13
GH2	β-mannosidase [EC:3.2.1.25]	4
GH11	Endo-1,4-beta-xylanase [EC:3.2.1.8]	4
GH67	α-Glucuronidase [EC:3.2.1.139]	1
CE1	Endo-1,4-beta-xylanase [EC:3.2.1.8]	2
CE4	Endo-1,4-beta-xylanase [EC:3.2.1.8]	3
CE6	para-Nitrobenzyl esterase [EC:3.1.1.-]	1
GH4	α-Galactosidase [EC:3.2.1.22]	1
GH127	α-Galactosidase [EC:3.2.1.22]	1
GH43	Arabinoxylan arabinofuranohydrolase [EC:3.2.1.55]	6
GH16	Endo-1,3–1,4-beta-glycanase ExoK [EC:3.2.1.-]	1
Cellulose degrading ability (9 putative CAZymes)		
GH3	β-Glucosidase [EC:3.2.1.21]	3
GH9	Endo-glucanase [EC:3.2.1.4]	3
GH94	Cellobiose phosphorylase [EC:2.4.1.20]	1
GH1	β-Glucosidase [EC:3.2.1.21]	1
GH8	Endo-glucanase [EC:3.2.1.4]	1
Pectic substrate and hemicellulose degrading ability (12 putative CAZymes)		
GH43	Endo-1,5-alpha-l-arabinosidase [EC:3.2.1.99]	5
GH51	α-l-Arabinofuranosidase [EC:3.2.1.55]	4
GH43	β-Galactosidase [EC:3.2.1.23]	2
GH2	β-Galactosidase [EC:3.2.1.23]	1
Others		
GH24	Lysozyme [EC:3.2.1.17]	1
GH73	N-acetylmuramoyl-l-alanine amidase [EC:3.5.1.28]	1
GH13	Starch synthase (maltosyl-transferring) [EC:2.4.99.16]	2

In total 71 putative CAZyme-encoding genes were annotated by dbCAN database; this table shows the merged results of both dbCAN and KEGG annotations

^a^The glycoside hydrolases/carbohydrate esterase families were predicted by use of the dbCAN database;

^b^Enzyme functions were predicted by use of the Kyoto Encyclopedia of Genes and Genomes (KEGG) database

### Identification of CAZyme gene clusters in the sequenced fosmid clones

By mapping Sanger end-tag sequences onto contigs, the genomic information of the individual fosmid inserts could be elucidated, which enabled to study the fibrolytic gene content and organization on the inserts. Bioinformatic analysis of 54 contigs revealed that 31 of these contigs possessed more than one CAZyme-encoding gene (Table 2). Many genes for CAZymes were found to be clustered in CAZyme gene clusters (CGC) that presumably bundle genes for functionally connected enzymes, which may act in a concerted manner for the degradation of substrate polymers to monomers (Additional file 1: Fig. S2). Some of the genes also encoded carbohydrate-binding domains, substrate binding proteins, transport systems and regulatory proteins. For example, contig 40 (GenBank Accession: OL125338, 36646 bps, assigned to fosmid clone BM1T-2 H7, Additional file 1: Fig. S2) carries five genes encoding putative GHs, including a GH67 α-glucuronidase, a GH3 β-glucosidase, a GH10 endo-β-xylanase and two GH43 endo-arabinanases. These enzyme families have the potential to degrade both the backbone and side chains of arabinoxylan.

### Similarity of the metagenome-encoded GHs with other proteins

**Fig. 1:**
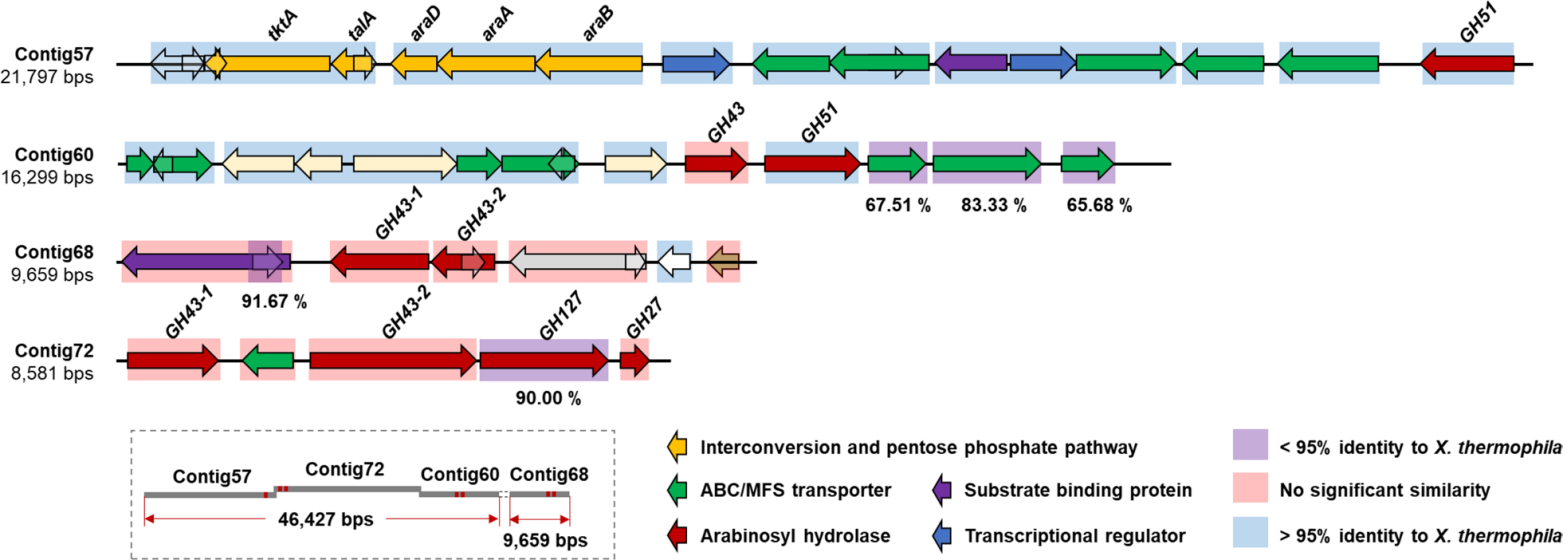
Genetic organization of putative gene clusters on an around 56 kb assembled metagenomic DNA fragment. The fragment encodes putative enzymes involved in arabinan and arabino-oligosaccharide degradation, substrate binding, transport, regulatory proteins and pentose metabolism. Red color: genes encoding putative arabinosyl hydrolases including family GH43, GH51, GH127 and GH27. Green color: genes encoding components of putative ABC-transporters/MFS transporters. Orange color: genes encoding major enzymes of the interconversion and pentose phosphate pathway. Blue background: more than 95% nucleotide sequence identity with *X. thermophila*. Purple background: lower than 95% nucleotide sequence identity with *X. thermophila*. Red background: no significant similarity. Schematic organization of four contigs in the box represents the assembled DNA fragment composed of contigs 68 (GenBank Accession: OL125336), 57 (GenBank Accession: OL125334), 72 (GenBank Accession: OL125337) and 60 (GenBank Accession: OL125335) of the shotgun sequencing assembly with around 125 bps overlapping regions in contigs 57, 72, 60, the presence of contig 68 in the DNA fragment was confirmed by PCR

It was possible to assemble an around 56 kb DNA fragment from four smaller shotgun sequencing contigs (contigs 68, 57, 72 and 60 with the GenBank accession number of OL125336, OL125334, OL125337, OL125335, respectively; the origin of all these contigs was from the sugar beet pulp enriched biogas fermentor community). By bioinformatic analysis, this DNA fragment showed the presence of a large cluster of genes encoding proteins putatively involved in pectic arabinan degradation, sugar-binding, transport and intracellular pentose metabolism (Fig. 1). Among them, seven putative arabinosyl hydrolases from GH51 and GH43, designated MC57GH51, MC72GH43-1, MC72GH43-2, MC60GH43, MC60GH51, MC68GH43-1, MC68GH43-2, respectively, were chosen for further characterization. The basic parameters of each protein are shown in Additional file 1: Table S4.

**Fig. 2:**
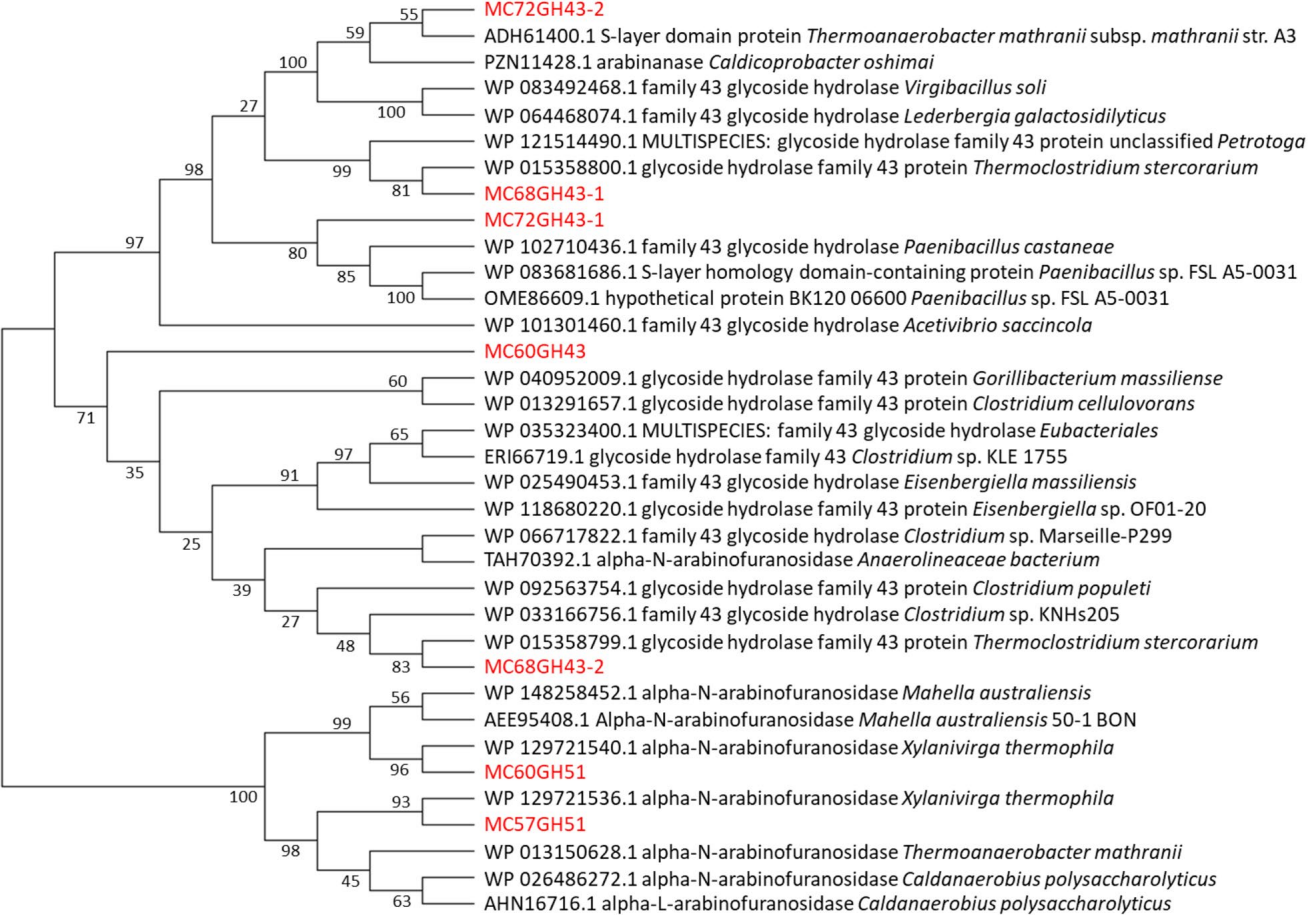
Phylogenetic analysis of seven putative arabinosyl hydrolase protein sequences. Multiple sequence alignments were performed with the algorithm MUSCLE against full-length sequences of GH43 or GH51 enzymes with each of their top 2–6 BLAST hits in the NCBI-nr database. Then a Neighbor-Joining (NJ) tree was calculated in MEGAX with bootstrap values from 500 replicates. Proteins from this study are colored in red, those from other sources in black. Database accession numbers and organisms are shown for each sequence

To visualize differences in sequences and phylogenetic relationships, a multiple amino acid sequence alignment was performed with each enzyme and its most closely related sequences (top two to six best hits in the NCBI-nr database), followed by the construction of a neighbor-joining tree (Fig. 2). In the phylogenetic tree, all putative GH43 family proteins are clearly separated from the GH51 proteins. The amino acid sequence identities between the putative GH43 enzymes ranged from 0 to 60.65% and the sequence identity of the two putative GH51 enzymes was 28.27%. Blast searches against the NCBI RefSeq database revealed a 74.94% amino acid sequence identity of MC72GH43-2 to an S-layer domain protein (ADH61400.1) from *Thermoanaerobacter mathranii*, while the other GH43 enzyme, MC72GH43-1, encoded on the same contig shared 63.68% identity at the amino acid sequence level with a GH43 protein (WP_102710436.1) from *Paenibacillus castaneae* (Fig. 2). Two GH43 proteins encoded on contig68 (MC68GH43-1 and MC68GH43-2) shared the closest similarity (81.35% and 84.31% amino acid sequence identity) with two GH43 (WP_015358800.1 and WP_015358799.1) from *Thermoclostridium stercorarium* (Fig. 2). Multiple alignments of the GH51 amino acid sequences from Contig60 and Contig57 with proteins from the RefSeq database revealed high identities of 97.42% and 99.59% with two protein sequences (WP_129721540.1 and WP_129721536.1, respectively) from *X. thermophila* (Fig. 2). Besides, a GH primary structure from MC60GH43 shared the highest identity (64.69%) with a GH43 protein (WP_040952009.1) from *Gorillibacterium massiliense* (Fig. 2).

### Expression and purification of putative arabinan-degrading enzymes

**Fig. 3:**
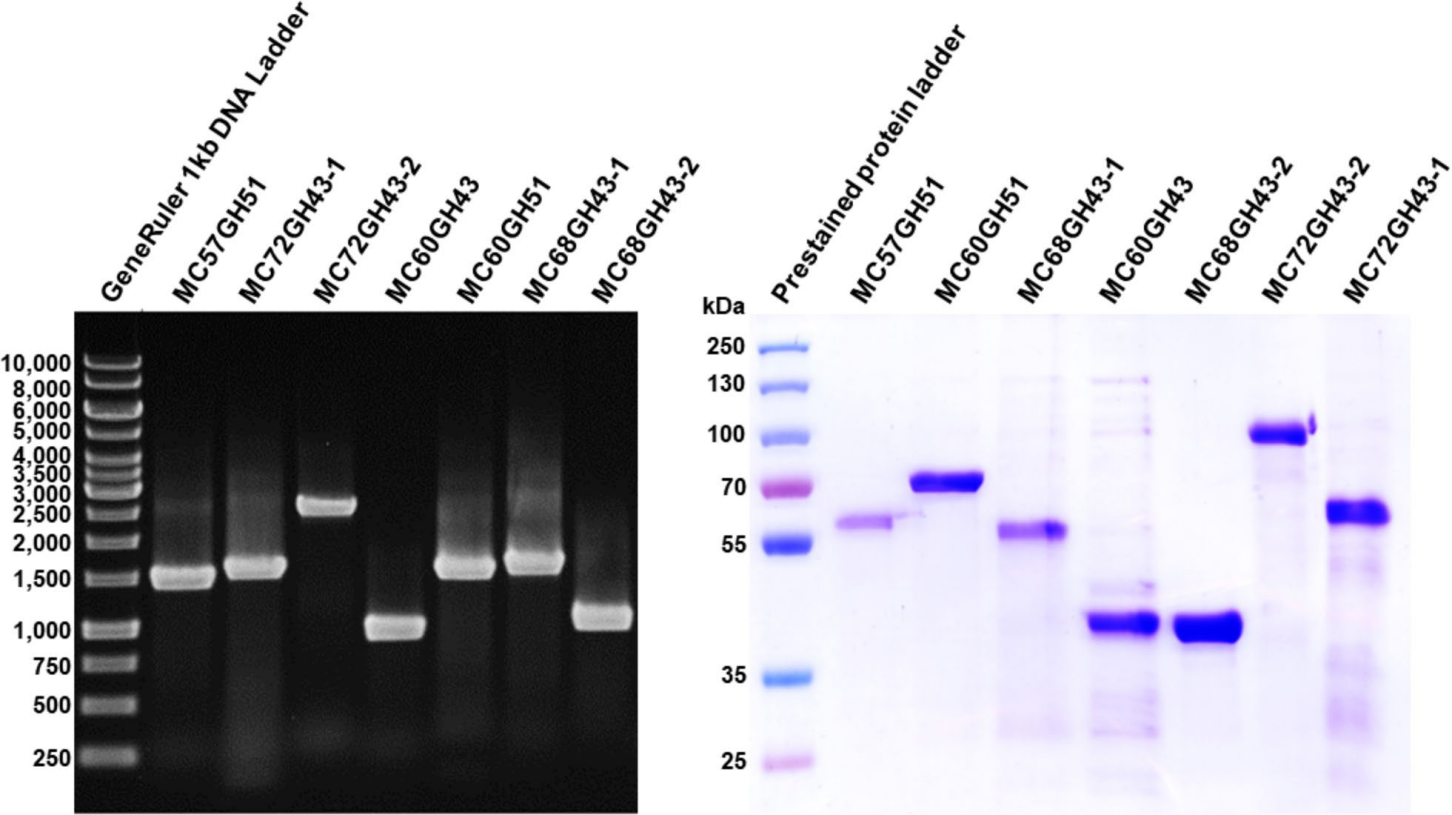
Cloning and expression of seven putative arabinosyl hydrolases from the enzyme families GH51 and GH43. The coding regions were identified in the sequence of the assembled DNA fragment shown in Fig. 1. **A** PCR amplification of the putative arabinosyl hydrolase-encoding genes was done using fosmid clone MOD18_Abn4 as a template. Lane 1: GeneRuler 1 kb, Lane 2: MC57GH51 (1,500 bps); Lane 3: MC72GH43-1 (1,407 bps); Lane 4: MC72GH43-2 (2,580 bps); Lane 5: MC60GH43 (960 bps); Lane 6: MC60GH51 (1,533 bps); Lane 7: MC68GH43-1 (1,575 bps); Lane 8: MC68GH43-2 (1,002 bps). **B** SDS-PAGE analysis of the purified recombinant arabinosyl hydrolases. The proteins were purified by immobilized metal affinity chromatography with a Ni-TED packed column and subsequent anion-exchange chromatography with a QFF column. Lane 1: prestained protein standards, Lane 2: MC57GH51 (56.65 kDa); Lane 3: MC60GH51 (58.40 kDa); Lane 4: MC68GH43-1 (58.80 kDa); Lane 5: MC60GH43 (36.71 kDa); Lane 6: MC68GH43-2 (39.23 kDa); Lane 7: MC72GH43-2 (95.88 kDa); Lane 8: MC72GH43-1 (52.18 kDa)

To explore the biochemical properties and the hydrolysis products formed by these putative arabinosyl hydrolases, their complete ORFs were amplified with simultaneous primer-based introduction of His_6_ tags at the C-termini of the target proteins and cloned in pET24c for heterologous expression in *E. coli* BL21 (Fig. 3A). The generated proteins with molecular masses of 56.65 kDa, 58.40 kDa, 36.71 kDa, 58.80 kDa, 39.23 kDa, 52.18 kDa or 95.88 kDa for MC57GH51, MC60GH51, MC60GH43, MC68GH43-1, MC68GH43-2, MC72GH43-1, and MC72GH43-2, respectively, were purified by His-tag-based affinity chromatography followed by anion-exchange chromatography. SDS-PAGE revealed pure protein bands with mobilities consistent with the calculated molecular masses of the hexa-histidine fusion proteins (Fig. 3B).

### Enzyme characterization

The optimum conditions of temperature and pH for the activity of each enzyme were determined by using *p*NP-AF (MC57GH51 and MC60GH51), DA (MC60GH43, MC68GH43-2, MC72GH43-2) or SBA (MC68GH43-1) as substrates. MC60GH43 showed maximum activity at 34.4 °C, while the maximum activities of all remaining enzymes were in the range of 49–56 °C. MC60GH43 maintained more than 60% activity in a temperature range of 25.0–41.9 °C. Other enzymes, such as MC68GH43-2 and MC72GH43-2, were more than 60% active at temperatures between 42.7 and 56.9 °C and 41.9–59.4 °C, respectively. The temperature ranges in which the activity exceeded 60% of the maximum activity of MC57GH51, MC60GH51 and MC68GH43-1 were between 45.6 and 70.6 °C, 38.1–63.1 °C and 37.6–57.5 °C, respectively. All enzymes preferred a slightly acidic pH and showed the highest activity between pH 4.5 and pH 6.5 (Table 4, Additional file 1: Fig. S3A–F). The stability of the enzymes was investigated under conditions resembling their temperature and pH optima by incubating them over a period of up to 24 h, and residual activities were determined via DNS assays with SBA or DA as substrate. The results revealed that MC68GH43-2 and MC60GH43 lost up to 30% of their initial activities over 24 h of incubation at their temperature and pH optima. The remaining four enzymes were more stable. They retained more than 80% activity in the same time period (Additional file 1: Fig. S3G).

**Table 4:** Optimal reaction conditions of arabinosyl hydrolases

Enzymes	pH _opt_	pH range	*T*_opt_ (°C)	*T*_range_(°C)	Substrates
MC57GH51	5.5	5.0–8.5	55.7	45.6–70.6	*p*NP-AF
MC60GH51	6.5	5.0–7.5	50.8	38.1–63.1	*p*NP-AF
MC68GH43-1	5.0	5.0–7.5	52.5	37.6–57.5	SBA
MC60GH43	4.5	4.5–6.5	34.4	25.0–41.9	DA
MC68GH43-2	5.0	5.0–5.5	49.3	42.7–56.9	DA
MC72GH43-2	5.5	4.5–8.0	55.0	41.9–59.4	DA

The enzymes (143.69 nM MC57GH51, 19.86 nM MC60GH51, 875.62 nM MC60GH43, 367.05 nM MC68GH43-2, 465.25 nM MC72GH43-2, 297.62 nM MC68GH43-1) were incubated in 200 µL reaction volume with 5 g L^−1^ the DA (debranched arabinan), SBA (Sugar beet arabinan) at pH 4.0 to 9.0 and between 25  °C and 80 °C for 30 min or in 50 µl reaction with 1 mM *p*NP-AF (*p*-Nitrophenyl-α-l-arabinofuranoside) for 10 min. Enzyme activities were determined by the reducing-end DNS assay (with DA/SBA as substrates) or *p*NP assay (with *p*NP-AF as substrate) by measuring of absorbance at OD_540_ and OD_405_, respectively, and represent mean values of triplicate assays. Borders for pH and temperature ranges were set to 60% of the highest activity.

### Activity of the arabinosyl hydrolases on polysaccharides and kinetic parameters

**Fig. 4:**
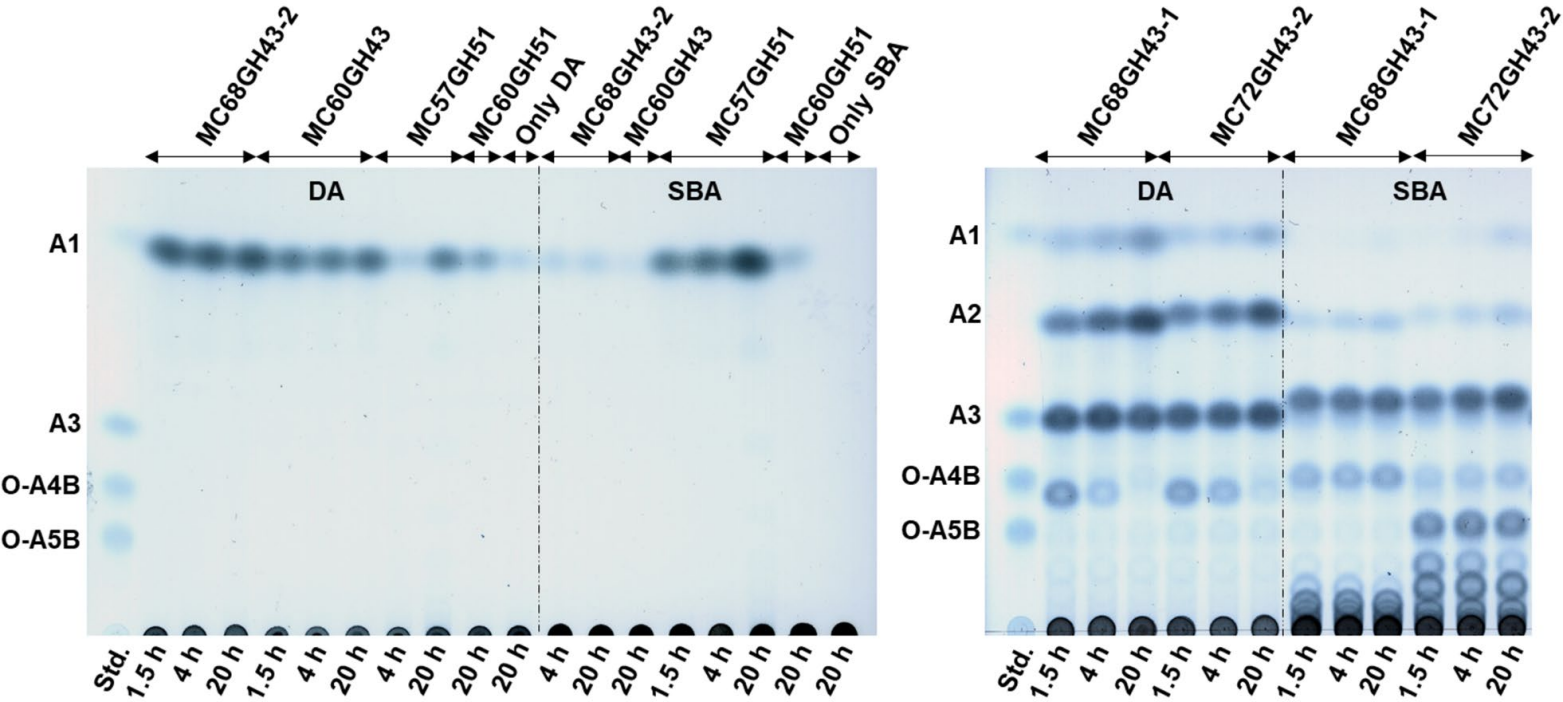
Analysis of hydrolysis product formation by exo-arabinofuranosidases **A** and endo-arabinanases **B** by TLC. Standard reactions were performed by incubating 5 g L^−1^ of SBA or DA with 211.83 nM MC57GH51, 19.69 nM MC60GH51, 457.64 nM MC60GH43, 2.64 µM MC68GH43-2, 130.37 nM MC72GH43-2 or 1.22 µM MC68GH43-1 at the enzymes’ optimal temperature and pH for different time periods. 10 µL samples were separated three times on TLC silica plates using chloroform:acetate:water (6:7:1, *v/v/v*) as a solvent

The kinetic parameters of each enzyme, including *K*_m_ and *V*_max_ values, were determined based on the Michaelis–Menten model. Only MC72GH43-1 did not show any detectable activity on all tested substrates. The remaining enzymes all exhibited activity against the artificial substrate *p*NP-AF and/or arabinan. Both MC57GH51 and MC60GH51 were not only active on *p*NP-AF, but also able to release arabinose from SBA and DA, with arabinose as the only hydrolysis product (Fig. 4A). This allows the conclusion that both GH51 enzymes are exo-acting α-l-arabinofuranosidases. The specific activity of MC57GH51 observed on SBA was 2.02 U mg^−1^, while its activity on DA was hard to detect with the DNS assay. MC60GH51 had a weaker activity than MC57GH51 with SBA as substrate (0.06 U mg^−1^) but revealed higher activity on DA (0.15 U mg^−1^) (Table 5). In comparison, MC68GH43-2 and MC60GH43 exhibited specific activities of 1.74 U mg^−1^ and 1.65 U mg^−1^, respectively, with DA as the substrate but only negligible activity with SBA and *p*NP-AF. The hydrolysis product released from both substrates was also only arabinose (Fig. 4A), which indicates that MC68GH43-2 and MC60GH43 are also exo-α-l-arabinofuranosidases. Regarding MC68GH43-1 and MC72GH43-2, no activity was found on *p*NP-AF, but they displayed a marked activity on SBA and DA (Table 5). TLC analysis of hydrolysis products of both MC68GH43-1 and MC72GH43-2 on different arabinan substrates revealed that the hydrolysis products included various AOSs and a small amount of arabinose (Fig. 4B). Therefore, both enzymes can be classified as endo-arabinanases. The hydrolysis products from DA after 20 h incubation were quite similar, including arabinose, arabinobiose and arabinotriose, whereas MC72GH43-2 released larger oligosaccharides from SBA than MC68GH43-1 (Fig. 4B). Finally, all these enzymes, including those with exo-cleavage activity towards SBA, in contrast to some exo-acting arabinofuranosidases involved in heteroxylan degradation, did not show any activity against wheat arabinoxylan, a polysaccharide with arabinose side-chain substitutions just like SBA.

**Table 5:** Specific activities and kinetic parameters of the characterized arabinosyl hydrolases on DA and SBA

	Specific activity^a^		Released soluble sugars^b^
Enzymes	SBA	DA	
MC57GH51	2.02 ± 0.11; *K*_m_ = 117.02, *v*_max_ = 52.67	ND	Arabinose
MC60GH51	0.06 ± 0.00; *K*_m_ and *v*_max_: ND	0.15 ± 0.01; *K*_m_ and *v*_max_: ND	Arabinose
MC60GH43	NA	1.65 ± 0.17; *K*_m_ = 83.05, *v*_max_ = 0.82	Arabinose
MC72GH43-1	NA	NA	NA
MC72GH43-2	3.81 ± 0.25; *K*_m_ = 14.98, *v*_max_ = 22.77	5.21 ± 0.21; *K*_m_ = 16.61, *v*_max_ = 18.27	AOS, Arabinose
MC68GH43-1	7.51 ± 0.31; *K*_m_ = 9.25, *v*_max_ = 2.96	2.39 ± 0.26; *K*_m_ = 11.04, *v*_max_ = 23.07	AOS, Arabinose
MC68GH43-2	ND	1.74 ± 0.02; *K*_m_ = 37.44, *v*_max_ = 14.02	Arabinose

K_m_ in g L^−1^, *v*_max_ in µmol min^−1^ mg^−1^, *K*_m_ and *v*_max_ were calculated by Microsoft Excel Solver based on enzyme activities at their optimal conditions with different substrate concentrations. ND means not determined due to insufficient activity or lack of detectable activity. *NA* means no activity

^a^Specific activity in µmol min^−1^ mg^−1^. The data show the mean values and standard deviations from three experiments

^b^Soluble sugars released, analyzed by HPAEC-PAD and TLC

*SBA* sugar beet arabinan, *DA* debranched arabinan, *AOS* arabino-oligosaccharides

Where possible, the enzymes’ kinetic properties were studied with DA or SBA as substrate. Among the exo-arabinofuranosidases, MC57GH51 only showed a detectable binding affinity for SBA, resulting in a *K*_m_ of 117.02 g L^−1^ and a *V*_max_ of 52.67 µmol min^−1^ mg^−1^ (Table 5, Additional file 1: Fig. S4G). In comparison, kinetic parameters of MC60GH43 and MC68GH43-2 could only be measured towards DA, which revealed that the former enzyme had a relatively lower binding affinity for DA than the latter enzyme (Table 5, Additional file 1: Fig. S4E, F). Both endo-arabinanases exhibited higher binding affinity for SBA than for DA (Table 5, Fig. Additional file 1: S4A-D).

### Cleavage specificity of the arabinosyl hydrolases towards oligosaccharides

**Fig.5:**
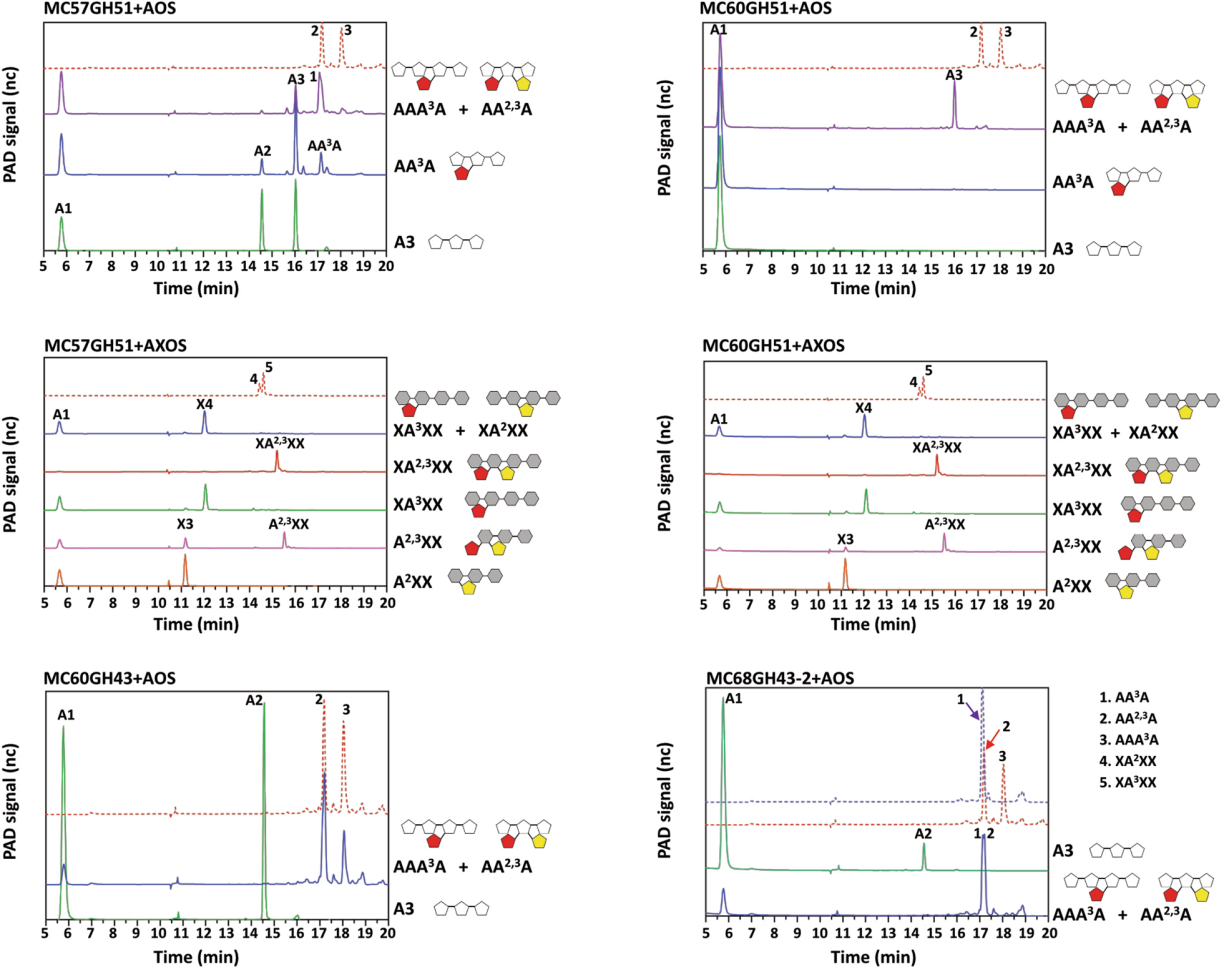
HPAEC-PAD analysis and structures of the hydrolysis products released from xylo-oligosaccharides and arabino-oligosaccharides. Standard reactions were performed in 10 µL reactions by incubating 0.1 µg of each enzyme with 2.5 mg mL^−1^ AOS or XOS at optimal temperature and pH for 12 h. The hydrolysis products were diluted 1:50 for HPAEC-PAD analysis. Grey hexagons represent xylopyranosyl residues, white pentagons represent arabinofuranosyl residues, red pentagons represent 1,3- linked arabinofuranosyl residues, yellow pentagons represent 1,2-linked arabinofuranosyl residues. The dashed-dotted lines represent the different standards

Various AOS and AXOS were incubated with the recombinant arabinosyl hydrolases for 12 h and the products obtained were analyzed by HPAEC-PAD. MC57GH51 and MC60GH51 were confirmed to be α-l-arabinofuranosidases that were able to cleave α-1,2, α-1,3, and α-1,5-linked arabinofuranosyl residues from purified arabino-/xylo- oligosaccharides, thus generating oligosaccharides with a lower degree of polymerization or with a simpler structure (Fig. 5A–D). MC60GH51 displayed higher activity than MC57GH51 on AOS since more arabinose was released by an equal amount of enzyme during the same incubation period (Fig. 5A, B). Even though both enzymes did not show any activity against the polysaccharide WAX, they could release arabinose from AXOS, including single 1,2- or 1,3-linked arabinofuranosyl residues, such as in XA^3^XX, XA^2^XX, A^2^XX, resulting in X4, X4, X3, respectively. They also displayed weak activity towards terminal double-substituted A^2,3^XX, releasing a minor amount of arabinose. However, no detectable activity was observed with internal double-substituted AXOS, such as XA^2,3^XX (Fig. 5C, D). In contrast, in agreement with the prominent activity of MC60GH43 and MC68GH43-2 against polymeric DA, a linear α-1,5-linked l-arabinofuranosyl backbone without side chains, these two enzymes were only able to release arabinose from arabinotriose and terminal unsubstituted 1,5-arabinosyl linkage in AAA^3^A (Fig. 5E, F).

### Synergistic action of arabinosyl hydrolases in arabinan degradation

**Fig. 6:**
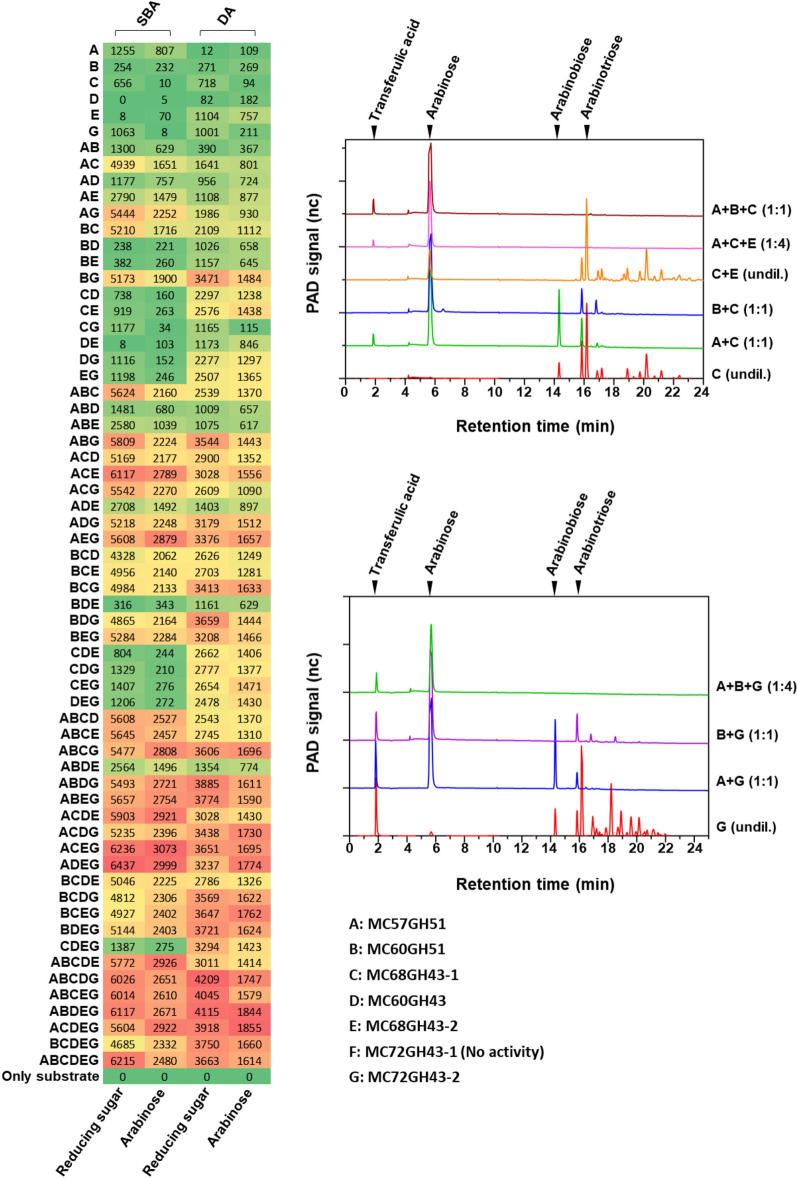
Hydrolysis of SBA or DA by different combinations of endo-arabinanases and exo-arabinofuranosidases. SBA or DA (5 g L^−1^) were incubated in 200 µL reactions containing 25 mM citrate–phosphate buffer pH 5.5 with single enzymes or enzyme combinations using enzyme concentrations of 75 nM (endo-arabinanases MC68GH43-1 and MC72GH43-2) and 150 nM (exo-arabinofuranosidases MC57GH51, MC60GH51, MC60GH43 and MC68GH43-2) at 50 °C for 24 h. **A** Evaluation of the yield of arabinose (HPAEC-PAD) and reducing sugar (DNS assay with arabinose as standard) released from SBA and DA in mg L^−1^. **B**, **C** HPAEC-PAD analysis of reactions carried out to identify the simplest enzyme combination which can fully hydrolyze SBA

To explore synergistic effects, the activities of single enzymes and numerous enzyme mixtures containing up to six different enzymes were tested using SBA and DA as substrates. The amount of arabinose released was quantified with HPAEC-PAD. Synergistic effects were observed especially with enzyme cocktails containing endo- as well as exo-enzyme activity, with maximum yields of 3073 mg L^−1^ arabinose for SBA degradation with enzyme cocktail MC57GH51, MC68GH43-1, MC68GH43-2, MC72GH43-2, and 1855 mg L^−1^ of arabinose for DA degradation with cocktail MC57GH51, MC68GH43-1, MC60GH43, MC68GH43-2, MC72GH43-2 (Fig. 6A). In the reactions performed with single enzymes, the final amount of arabinose released was only 0.16–26% (with SBA) and 5.54–44.66% (with DA) of that compared with the maximum yields reached with the enzyme mixtures just mentioned. It is noteworthy that endo-arabinanase mostly released arabino-oligosaccharides from SBA which resulted in a high concentration of reducing sugar but a low amount of arabinose observed in the hydrolysis products (Fig. 6A). Moreover, the amount of arabinose released from different arabinan substrates was not always significantly boosted by any arbitrary combination of endo-arabinanase and exo-arabinofuranosidase. For SBA degradation, the combination of an endo-arabinanase, either MC68GH43-1 or MC72GH43-2, with the exo-arabinofuranosidase(s) MC57GH51 and/or MC60GH51 revealed a clear synergistic effect. On the other hand, when these endo-arabinanases (MC68GH43-1 or MC72GH43-2) were combined with MC60GH43 and/or MC68GH43-2, the yield of arabinose from SBA remained relatively low. Compared with MC60GH43 and MC68GH43-2, which are only capable of cleaving 1,5-arabinosidic bonds, MC57GH51 and MC60GH51 have a broader cleavage specificity and higher cleavage efficiency towards different types of side chains only present in SBA but not in DA. For DA hydrolysis, in contrast, the combination of the exo-arabinofuranosidase MC60GH43 and/or MC68GH43-2 with one of the endo-arabinanases dramatically improved the reaction. The results also suggest that the synergism of the enzymes characterized here was mainly dependent on which exo-active enzyme was chosen rather than on which endo-arabinanase was included in the assay reaction.

Different enzyme combinations were tested to find out the most efficient one with regard to the arabinose yield. A cocktail composed of MC57GH51, MC68GH43-1 and MC60GH51 efficiently degraded SBA to arabinose. MC68GH43-2 was an efficient alternative to MC60GH51, and MC72GH43-2 was an efficient alternative to MC68GH43-1 (Fig. 6B, C). Interestingly, for certain efficient enzyme combinations, addition of further enzymes not only did not increase the arabinose yield, but on the contrary even led to a slight decrease in the arabinose yield. For example, with enzyme cocktail MC57GH51, MC60GH51, MC68GH43-1, MC60GH43, MC68GH43-2, MC72GH43-2 the yield decreased compared with cocktail MC57GH51, MC68GH43-1, MC68GH43-2, MC72GH43-2 (Fig. 6A). This may be explained by the release of high amounts of mono- and oligo-saccharides leading to an end-product inhibition as described for some enzymes [26, 27].

### Bioinformatic identification of additional arabinan-degrading enzymes

To identify further genes that could play a role in arabinan degradation, we inspected the genetic context close to the genes for the characterized arabinosyl hydrolases on the assembled ~ 56 kb metagenomic DNA fragment. All 48 ORFs on this fragment were annotated by using the KEGG and dbCAN analysis tools. Besides the genes for the six characterized and one inactive arabinosyl hydrolases, we found genes for two putative CAZymes from the GH127 and GH27 families, which could also be related to arabinan degradation. In addition, the assembled DNA fragment also carries 22 further genes that encode sugar transport components, regulatory proteins and proteins involved in pentose and glucuronate interconversions and pentose phosphate pathway, including transketolase, transaldolase, fructose-6-phosphate aldolase, ribulokinase, l-arabinose isomerase and l-ribulose-5-phosphate 4-epimerase (Fig. 1). It is noteworthy that the ORF encoding a substrate binding protein was predicted to contain a lipoprotein signal peptide and is assumed to be a lipoprotein. Such proteins usually anchor to the surface of the cytoplasmic membrane. A large number of the ORFs showed the highest similarities (99–100% nucleotide sequence identity) with genomic DNA sequences from *X. thermophila* (Fig. 1). Therefore, we speculate that this assembled metagenomic DNA fragment, which was isolated from the metagenome of a lab-scale biogas fermenter fed with maize silage, may originate from *X. thermophila*, a largely uncharacterized *Clostridiales* member from the phylum *Bacillota (*syn. *Firmicutes*), or from a closely related organism. The results presented here suggest the involvement of the pentose and glucuronate interconversions and pentose phosphate pathway in pentose utilization of *X. thermophila.*

## Discussion

The enrichment of microbial communities on recalcitrant lignocellulolytic biomass, such as maize silage, sugar beet pulp or wheat straw, can shape the communities and enrich genetic information related to the decomposition of such biomass [23, 28]. Commonly, *Firmicutes* and *Bacteroidetes* are dominant phyla in biogas digesters [29], but the ratio of these two phyla is affected by various factors [30]. 16S rRNA amplicon sequencing results revealed that the bacterial structure in fermenter samples T1T2 and MOD18, which underwent anaerobic enrichment through feeding with SBP, mainly included *Bacillota (syn. Firmicutes)*, *Thermotogae* and a small fraction of *Bacteroidetes* (Table S1). These phyla have repeatedly been reported as predominant phyla involved in the thermophilic utilization of complex carbohydrates [31, 32]. The microbial consortia T1T2 and MOD18 were chosen for the construction of metagenomic libraries and search for polysaccharide-degrading CAZy-encoding genes because they displayed rapid degradation of SBP at elevated temperature (55 °C and 50 °C, respectively).

Despite the enrichment on the pectin-rich substrate SBP, unexpectedly, no fosmid clone was able to degrade pectin with the screening assay used in our study. This could have been caused by a relatively low abundance of pectinase producers in the samples used for DNA extraction and metagenomic library construction or a poor coverage of the metagenome sequences represented in the libraries, leading to a low probability of finding clones with this activity. Screening even more clones could help to resolve this problem. Another reason may be that not all cells from a complex microbial community are lysed equally well during the DNA extraction procedure. Further possible reasons are poor gene expression of some genes in the library host, the need to simultaneously express more than one enzyme to achieve the desired screening phenotype, or poor efficiency of detection of pectinases by the functional screening assay used, which also have been bottleneck issues in other metagenomic studies [23]. To overcome the barriers of ineffective cloning and expression of foreign genes in *E. coli*, various expression vectors [33, 34] and alternative hosts [24, 35, 36] have been developed, but their application requires substantially increased manpower and resources.

For the functional screening of metagenomic libraries, in this study, we developed CPH substrates by labeling polysaccharides with chlorotriazine dyes [37]. The CPH substrates were prepared in several colors, which enables the development of a high-throughput assay system by combining different color-labeled substrates in single wells of reaction plates. Thus, multiple enzymes from the same fosmid clone or single enzymes with side activities can be detected in a single well. This novel screening approach, however, has certain limitations. Acetyl-ester modifications, for example, are absent from CPH/ICB substrates, but are prevalent on xylan. This is due to the high pH employed during substrate production, which causes ester linkages to be hydrolyzed. Additionally, because of the steric hindrance of cleavage sites, the CPH and ICB substrates are not suited for certain accessory enzymes of polysaccharide decomposition, such as exo-acting enzymes. As a result, alternative approaches for assaying these enzymes may be required [37]. However, since genes for polysaccharide degradation are often clustered in genome sequences, such accessory enzymes can be identified with bioinformatics tools in the vicinity of other genes of polysaccharide hydrolysis and utilization.

To fully characterize the fosmid clones identified by functional assays, their recombinant fosmid inserts were sequenced using a “pooled strategy” as described by Lam et al*.* [38] and Sanger end sequencing was used to assign contig sequences to specific clones and their fosmids. Besides single genes putatively encoding CAZymes, functional annotation by the dbCAN database also revealed various gene clusters containing CAZyme genes, which could be involved in the saccharification of complex heteropolysaccharides. It is noteworthy that contig40 (36,646 bps), which was assigned to fosmid clone “BM1T-2 H7” (Additional file 1: Fig. S2), carried five putative GH encoding genes involved in arabinoxylan degradation. Two of them, encoding putative GH3 and GH10 enzymes, were also identified in a different metagenomic study of a thermophilic methanogenic digester sample and further proved to have xylosidase and xylanase activity, respectively [39].

Phylogenetic analyses of the seven arabinosyl hydrolase proteins encoded on the large assembled metagenomic DNA fragment studied in this work revealed that the most closely related proteins were from *Clostridium* species, *Xylanivirga thermophila* and *Caldicoprobacter oshimai*. Blast analysis suggests that the metagenomic DNA fragment may originate from a strain of the recently described species *X. thermophila*, with *Caldicoprobacter faecalis* DSM 20678^ T^ as its closest relative (89.9% 16S rRNA identity) [40].

All the proteins characterized in our study do not contain predicted signal peptides, which indicates that they are putatively intracellular proteins. However, two enzymes were found to be endo-arabinanases with hydrolytic activity on arabinose polymer substrates. SignalP predicts only conserved signal sequences of proteins transported predominantly by the Sec or Tat pathways [41]. However, non-canonical secretion by the natural host cannot be excluded since some proteins are apparently secreted with so far unknown export mechanisms which sometimes are species-specific or limited to only a few proteins [42, 43]. For example, a study focused on the secretome of *B. subtilis* showed that the genome-based annotations mirrored the real composition of the extracellular proteome to only 50% [44]. In case that the arabinanases of this study are really intracellularly localized in their native host, they could be active on relatively long arabino-oligosaccharides transported into the cell. Arabinofuranosidases are sometimes extracellular, but this type of enzyme is commonly also found intracellularly where they participate in the hydrolysis of arabinose-containing oligosaccharides after their internalization into the cytoplasm or periplasm [45, 46].

The biochemical characterization results of the arabinosyl hydrolases of our study, which have similar temperature and pH optima, provide indications about the physiological characteristics of the natural host strain. The pH optima of the characterized enzymes range from pH 4.5 to 6.5, which is not unusual for intracellular enzymes but argues against their extracellular localization in the alkaline growth environment of *X. thermophila* at pH 8.0 [40]. Except MC60GH43_,_ all enzymes described in our study showed optimal activity at temperatures of around 50 °C, which is close to the temperature optimum of *X. thermophila* [40] and fits the moderately thermophilic surroundings in the biogas fermenter used for our study (50 °C). Besides, all the arabinosyl hydrolases are quite stable under their conditions of maximal activity at around 50 °C, which is a favorable feature for their possible application in enzyme cocktails composed for the degradation of recalcitrant substrates.

dbCAN analysis of the two endo-arabinanases (MC68GH43-1, MC72GH43-2) showed that both enzymes belong to the GH43_4 subfamily. This subfamily and subfamilies GH43_5, GH43_6 are the only ones predicted to exhibit endo-α-l-arabinanase activity (EC 3.2.1.99), which is in agreement with the characterization results from our study. Similar to MC68GH43-1 and MC72GH43-2, arabinanases from *T. petrophila* [47], *B. subtilis* [48] and *C. polysaccharolyticus* [49] were also assigned to the GH43_4 subfamily and showed endo-arabinanase activity. The product patterns of these enzymes for DA degradation are similar. The main end products of MC68GH43-1 and MC72GH43-2 were arabinose, arabinobiose and arabinotriose, whereas the hydrolysis products of the arabinanases from *B. subtilis* and *C. polysaccharolyticus* on DA consisted primarily of arabinose and arabinobiose [48, 49]. With SBA as substrate MC72GH43-2 preferably released oligosaccharides with a higher degree of polymerization than MC68GH43-1. Regardless of the differences in cleavage products, the generated oligosaccharides were easily digested by exo-acting arabinofuranosidases according to our results about enzyme synergism during substrate degradation (Fig. 6).

Both MC72GH43-2 and MC68GH43-1 revealed a relatively strong binding affinity for SBA. The difference in arabinan substrate binding may be related to the specific module architecture of both enzymes. MC68GH43-1 contains a GH43 domain and a GH43_C domain, while MC72GH43-2 additionally contains a laminin_G_3 domain. A laminin_G_3 domain was reported to play a role in substrate binding of an arabinofuranosidase from *Ruminococcus josui*, specifically with a branched arabinofuranosyl residue of an arabino-oligosaccharide [50]. Regarding the exo-arabinofuranosidases, MC72GH43-1 also displayed a multi-modular structure, being composed of a GH43 module and a module associated with some GH43 modules, GH43_C2 (Pfam designation PF17851, beta-xylosidase C-terminal concanavalin A-like domain). The function of the GH43_C2 module is unknown. The other four arabinofuranosidases exhibited single-module structures, with only either a single GH51 or a single GH43 module. Regarding arabinan degradation, two GH51 enzymes displayed high activity against SBA, but relatively low activities against DA, while the two GH43 enzymes MC60GH43 and MC68GH43-2 only showed activity against DA. DA has a backbone of α-1,5-linked arabinofuranosyl residues with a very low degree of substitution, whereas in SBA, 60% of the *O*-3 positions and a lower frequency of the *O*-2 positions of the linear arabinan backbone are substituted with arabinofuranose residues [51]. This demonstrates that MC60GH43 and MC68GH43-2 efficiently degrade α-1,5-arabinofuranosyl linkages of the arabinan backbone, while MC57GH51 and MC60GH51 had the ability to cleave off the side chains of SBA. If the latter enzymes can remove arabinose substituents linked to both *O*-3 and *O*-2 of the backbone residues of the polymeric SBA cannot be deduced from the experimental data, but considering the high *O*-3 substitution degree of SBA (see above) in context with their activity on branched arabino-oligosaccharides (Fig. 5) it seems plausible that at least *O*-3 substitutions can be removed.

With arabinosylated arabino- and xylo-oligosaccharides as substrates, MC57GH51 and MC60GH51 were not only able to remove arabinosyl substituents at *O*-2 or *O*-3 positions of both AOS and XOS, but also can cleave off terminal *O*-5 arabinosyl residues, which is in consistency with the polysaccharide degradation results. The activities of both enzymes against internally di-arabinosylated XA^2,3^XX were negligible. In comparison, with terminally double-substituted side chains in A^2,3^XX and internally di-arabinosyl side chains in AA^2,3^A, the enzymes displayed activity, which is similar to the GH51 isolated from *A. mesophilus* [52]. All these enzymes, including MC57GH51 and MC60GH51, did not show any activity with WAX, despite the presence of the same arabinosyl side-chain substitution in this substrate polymer. The results with the XOSs and WAX are in line with the idea that arabinofuranosyl di-substitutions that modify the xylan backbone are recalcitrant to cleavage by arabinofuranosidases [53]. On the other hand, our results with the internally di-substituted AOS substrate with its arabinose-based backbone demonstrate that the backbone structure, which in the case of AOS is composed of arabinose, can be of major importance for the activity of these enzymes.

It is noteworthy that two more genes encoding putative arabinanases/arabinofuranosidases from the families GH127 and GH27 were also found on the ~ 56 kb large metagenomic DNA fragment that carries the arabinosyl hydrolase genes investigated in this study. The GH127 and GH27 families are not as well studied as the other arabinanase/arabinofuranosidase-containing families. The first GH127 β-arabinofuranosidase was found from *Bifidobacterium longum*, which was able to cleave glycoprotein-derived β-arabinofuranose residues [54]. Another study further showed that GH127 can release special arabinosyl groups in pectic structural elements, which include dimeric side chains containing a terminal β-arabinofuranose [55, 56]. By contrast, the GH27 family includes β-arabinopyranosidase activity with the potential for degradation of terminal β-arabinopyranose residues, which is exemplified by a characterized GH27 from *C. polysaccharolyticus* [49]. Unfortunately, the specific substrates for further analysis, *p*NP-β-l-arabinopyranoside and *p*NP-β-l-arabinofuranoside, were not available to us for characterization of these activities.

The annotated ORFs (KEGG Blast KOALA annotation) on the ~ 56 kb large metagenomic DNA fragment, as argued above, putatively originates from a strain of *X. thermophila.* This DNA fragment was functionally annotated to encode not only the characterized and additional putative arabinosyl hydrolases, but also genes for sugar transport, regulatory proteins, as well as the enzymes of the pentose interconversions and pentose phosphate pathway, including l-arabinose isomerase, l-ribulose-5-phosphate 4-epimerase, transketolase, transaldolase, l-ribulokinase, which indicates the functional clustering of genes for pentose-liberating together with pentose-metabolizing enzymes. Therefore, considering the demonstrated functions of the CAZymes characterized in this study, as well as other putative gene functions deduced from ORF annotations, we suggest that the enzymes encoded on the ~ 56 kb large metagenomic DNA fragment studied here play an important role in pentose liberation and metabolism in *X. thermophila*. The endo-arabinanases MC68GH43-1 and MC72GH43-2 could be involved in the cleavage of arabinan polysaccharides, typically found in pectic substances as side chains of RGI, into arabino-oligosaccharides. Since their primary structures do not contain signal peptides, it is unclear if they are secreted or perhaps could be released via a non-canonical secretion mechanism. Alternatively, in the natural habitat, other polysaccharide degraders may contribute to the extracellular enzymes needed for polysaccharide cleavage to oligosaccharides. After internalization of arabinan-derived oligosaccharides, intracellular enzymes in *X. thermophila*, such as MC57GH51, MC60GH51, MC60GH43 and MC68GH43-2, may further hydrolyze the arabino-oligosaccharides to yield arabinose before the pentose is catabolized in the pentose interconversions and pentose phosphate pathway.

## Conclusions

The arabinosyl hydrolases studied here, which were all encoded on an assembled metagenomic DNA fragment, revealed complementing catalytic capabilities and strong synergistic effects towards sugar beet arabinan degradation. These enzymes, together with further predicted functions encoded on the same fragment, including substrate binding, transport, pentose interconversions and the whole pentose phosphate pathway, apparently contribute to the degradation and utilization of arabinose-containing substrates in *X. thermophila*. The arabinosyl hydrolases described here may be useful for the design of efficient enzyme cocktails for the biotechnological valorization of sugar beet pulp or arabinan extracted from this agricultural residue.

## Methods

### Construction of metagenomic library

Eleven different fermenter samples with microbial communities from a thermophilic biogas fermenter residue were inoculated into 10 mL GS2-based medium as described by Rettenmaier et al. with 1% SBP as substrate to test their ability to degrade SBP [57]. Only four samples were found to degrade SBP completely and rapidly within 10 days. Two of them (named T1T2 and MOD18), which displayed rapid SBP degradation at elevated temperatures (55 °C and 50 °C, respectively), were chosen for metagenomic library construction with the CopyControl^™^ Fosmid Library Production Kit (Epicentre Technologies), with pCC1FOS fosmid as a vector and EPI300^™^-T1R Phage T1-resistant *E. coli* strain as host.

For metagenomic DNA extraction, a CTAB-chloroform:isoamyl alcohol protocol was adopted as described by Lebuhn et al. since it performed well in reducing humic substance contamination [58]. Following that, the metagenomic DNA was mechanically sheared into around 40-kb fragments by using a Hamilton syringe. The size of the resulting DNA fragments was estimated by pulsed-field gel electrophoresis with a BioRad Chef-DRIII System (initial time: 1 s, final time: 25 s, run time: 15 h, volt: 6), using a 40-kb fosmid insert DNA as a reference. The fragmented DNA was further cloned into pCC1FOS fosmid by using the CopyControl™ Fosmid Library Production Kit as recommended by the manufacturer. In addition to the newly constructed libraries, 7779 fosmid clones from existing libraries, which were constructed in either pCC1FOS fosmid or pCT3FK fosmid, were also subjected to further functional screening.

### 16S rRNA amplicon sequencing

All samples for 16S rRNA amplicon sequencing were prepared by centrifugation of 1 mL samples of cultures at 13,400 rpm for 5 min, before resuspension of the pellets in 600 µL RNAlater and storage at -20 °C for sequencing. Amplicon library preparation and high-throughput sequencing of the V3–V4 gene region of 16S rRNA at ZIEL-Core Facility Microbiome/NGS (TU Munich, Germany) were performed as described by Reitmeier et al. [59]. Briefly, the sequencing was carried out by a two-step PCR amplification procedure (15 cycles for each PCR program) with 24 ng isolated DNA as a template [60]. After purification with AGENCOURT AMPure XP beads (Beckman Coulter), sequencing was performed in paired-end mode (PE275) with purified libraries that were spiked-in with 20% (v/v) PhiX DNA in an Illumina MiSeq system prepared according to the manufacturer’s instruction [61].

### Preparation of CPH and ICB substrates

All the chromogenic substrates used in our study were produced in-house by first dyeing polysaccharides with chlorotriazine dyes (reactive blue 4/120/19, from Santa Cruz Biotechnology, Heidelberg, Germany), followed by covalent crosslinking with 1,4-butanediol diglycidyl ether (from Santa Cruz Biotechnology, Heidelberg, Germany) to obtain insolubilized chromogenic polysaccharides. Polysaccharides/plant biomass used for chromogenic substrates preparation included oat spelts xylan, CMC (both from Sigma-Aldrich, Munich, Germany), SBA (sugar beet arabinan; from Megazyme, Wicklow, Ireland) and arabinoxylan (from ASA Spezialenzyme GmbH, Wolfenbüttel, Germany). SBP used in our study was washed with boiling water three times to remove free monosaccharide residues. After drying, it was further ground into small particles for ICB substrate preparation. The production of CPH and ICB substrates was performed according to the method of Kračun et al*.* [37]. The applicability of the CPH and ICB substrates was further tested by using commercial enzymes before screening.

### High-throughput functional screening of fosmid libraries

For metagenomic library screening, fosmid clones from − 80 °C glycerol stocks were inoculated into 1 mL fresh LB medium supplemented with 12.5 μg mL^−1^ of chloramphenicol and 0.1% arabinose and incubated on an orbital shaker (300 rpm) at 37 °C. After about 16 h, the cultures were subjected to three freeze (− 80 °C)/thaw cycles to disrupt the cells. Using a 96 Channel Portable Electronic Pipette (INTEGRA Biosciences AG, Switzerland), aliquots of 150 µL were transferred into 96-well plates containing pre-distributed single chromogenic substrates or different combinations of substrates. 25 mM citrate–phosphate buffer pH 7.0 was applied to each reaction, resulting in a final volume of 200 µL. The plate was sealed using an adhesive PCR membrane and incubated overnight at 37 °C without shaking. Positive clones were identified by the change of color in the supernatant.

### Pool-sequencing strategy for sequencing of fosmids

Recombinant fosmids from clones exerting activity on one or more of the chromogenic substrates were sequenced with a combined Illumina–Sanger sequencing strategy as described by Lam et al*.* [38]. For this, the fosmids were isolated from 5 mL overnight cultures of *E. coli* EPI300 clones using the Monarch® Plasmid Miniprep kit (NEW ENGLAND BioLabs, Massachusetts, USA). Two library pools were created based on the fosmid backbones, comprising either 40 pCC1FOS- or 15 pCT3FK-based recombinant fosmids. Each fosmid pool contained equal concentrations of each fosmid and had a final total DNA concentration of 20 µg mL^−1^. Preparation of sequencing libraries was performed by using Illumina DNA Prep Kit (Illumina Inc, USA, Cat. No. 20018705) according to the suppliers’ manual and sequencing was performed using an Illumina NovaSeq System at ZIEL-Core Facility Microbiome/NGS. The fosmid pools were sequenced with approximately 50,000-fold coverage. After sequencing, the obtained reads were assembled by using Unicycler v0.4.8.0. The removal of vector backbone and contaminating *E. coli* genomic DNA sequences was performed manually by BLAST analysis of the obtained contigs against the NCBI database.

In order to assign the contigs to individual fosmids, the terminal 1000 bp of each fosmid insert was sequenced with Sanger technology at Eurofins Genomics (Ebersberg, Germany). The sequencing primers used were standard pCC1FOS fosmid and pCT3FK fosmid forward and reverse primers, respectively, which are listed in Table S5. The obtained end-tags were then used to query the pooled, assembled sequencing results.

The open reading frames (ORFs) encoded on the contigs were identified by using the Clone Manager (Sci Ed Software LLC, Westminster, CO, USA) ORF searching function. The identification of ORFs for CAZymes and of CAZyme gene clusters (CGC) was performed by searching against the dbCAN database with default parameters. The predicted amino acid sequences were submitted to the KEGG database for further functional prediction (*E*-value < 1.0E-5). Signal peptides were predicted by SignalP-5.0. Conserved domains of the enzymes were predicted by the Pfam database. Molecular masses and isoelectric points (pI) were predicted by using the Clone Manager software.

### Gene expression and protein purification

To better understand biochemical properties of pectic arabinan-degrading gene cluster derived from fosmid library, in this study, a total of 7 genes distributed in an assembled DNA fragment, which assigned to fosmid clone MOD18_Abn4, were subjected to prokaryotic cloning and functional analysis, including MC68GH43-1 (abbreviation for the first GH43 from metagenomic contig 68), MC60GH43, MC72GH43-1, MC72GH43-2, MC57GH51, MC60GH51 and MC68GH43-2. All genes were amplified from the fosmid clone MOD18_Abn4 using primers which introduce six histidines at the C-terminus of the protein (primers are listed in Additional file 1: Table S5). The PCR amplicons were then cloned in *NdeI*/*XhoI* linearized pET24c vector by Gibson Assembly (New England Biolabs, Massachusetts, USA). Transformation and induction, purification and quantification of the recombinant proteins were performed following the same procedures as described in our earlier study [52], except a lower concentration of IPTG of 0.05 mM and a shorter incubation time of 6 h at 30 °C was applied to protein MC60GH51 and MC68GH43-2 induction to avoid inclusion body formation.

### Enzyme assays

The enzyme activity was measured with *p*-nitrophenyl-α-l-arabinofuranoside (*p*NP-AF, Megazyme), various polysaccharide substrates (Megazyme, Wicklow, Ireland), including insoluble wheat arabinoxylan, arabinoxylan for reducing sugar assay (both from wheat flour), sugar beet arabinan (SBA; monomer composition: arabinose: galactose: rhamnose: galacturonic acid: other sugar = 69: 18.7: 1.4: 10.2: 0.7), and debranched arabinan (DA; monomer composition: arabinose: galactose: rhamnose = 71: 26: 3), and oligosaccharides (Megazyme, Wicklow, Ireland), including O-ATR (Arabinotriose), O-A4B (3^2^-α-l-arabinofuranosyl-(1,5)-α-l-arabinotriose), O-A5B MIX (2^2^,3^2^-di-α-l-arabinofuranosyl-(1,5)-α-l-arabinotriose plus 3^2^-α-l-arabinofuranosyl-(1,5)-α-l-arabinotetraose, O-AX3 (2^3^-α-l-arabinofuranosyl-xylotriose), O-XAXX MIX (3^3^-α-l- plus 2^3^-α-l-arabinofuranosyl-xylotetraose (XA^3^XX/XA^2^XX) mixture), O-XA3XX (3^3^-α-l-arabinofuranosyl-xylotetraose), O-A2X3 (2^2^, 3^2^-di-α-l-arabinofuranosyl-xylotriose), O-XA23XX (2^2^, 3^2^-di-α-l-arabinofuranosyl-xylotetraose) (the nomenclature and the structure of oligosaccharides are guided by Fauré et al*.* and Liu et al.) [52, 62]. All insoluble substrates were washed with Milli-Q water to remove the soluble reducing sugars before starting the enzyme reaction.

Chromogenic assays with *para-*nitrophenyl (*p*NP)-coupled substrates (*p*NP assay) were performed in 50 µL reaction mixtures containing 25 mM citrate–phosphate buffer (citric acid, Na_2_HPO_4_, 50 mM NaCl) and 1 mM *p*NP-AF as substrate and appropriately diluted enzyme. Reactions were terminated after 10 min by adding two volumes of 1 M Na_2_CO_3_, which was followed by measuring the absorbance at 405 nm using a 230ND-1000 Spectrophotometer (NanoDrop^®^). With DA or SBA as substrate, the reducing ends released by enzymatic cleavage were quantified using the 3,5-dinitrosalicylic acid-reducing sugar assay (DNS assay), using arabinose as standard [63]. For this, the enzymes were incubated with 5 g L^−1^ DA or SBA in a 200 µL volume containing 25 mM citrate–phosphate buffer. After 30 min to 2 h incubation, 50 µL of the hydrolysis products were mixed with 75 µL DNS reagent (10 g L^−1^ DNSA; 200 g L^−1^ K–Na-tartrate; 10 g L^−1^ NaOH; 0.5 g L^−1^ Na-sulfate; 2 g L^−1^ phenol) and incubated at 95 °C for 10 min. The products were then transferred into 96-well plates for absorbance measurement at 540 nm wavelength. All assays were performed in triplicates.

The influence of temperature and pH on enzyme activity was investigated in the temperature range between 25 °C and 90 °C and the pH range between pH 2.0 and pH 9.0 (pH adjusted at 50 °C). The substrates used here were *p*NP-AF for MC57GH51 and MC60GH51, DA for MC60GH43, MC68GH43-2 and MC72GH43-2, SBA for MC68GH43-1. The resistance of the enzymes against thermoinactivation was explored by incubating enzymes at their optimal temperature and pH for various time spans before measuring residual activity against SBA (MC57GH51, MC60GH51, MC68GH43-1) or DA (MC60GH43, MC68GH43-2, MC72GH43-2).

The specific activity of each enzyme was measured by incubating the enzymes at concentrations between 45 nM and 1.07 µmol with 5 g L^−1^ SBA or DA at their optimum temperature and pH with shaking (600 rpm) for different time periods (30 min, 1 h and 2 h). The released reducing sugars were quantified with the DNS assay. All assays were performed in triplicates. One unit of enzymatic activity was defined as the amount of enzyme required to release 1 µmol of l-arabinose equivalent per minute.

The kinetic parameters of each enzyme were investigated at optimal working conditions of pH and temperature in a reaction volume of 200 µL. The substrates SBA or washed DA were tested at different concentrations (50 g L^−1^, 45 g L^−1^, 40 g L^−1^, 35 g L^−1^, 30 g L^−1^, 25 g L^−1^, 15 g L^−1^, 10 g L^−1^, 7.5 g L^−1^, 5 g L^−1^, 2.5 g L^−1^, 1 g L^−1^). Three different concentrations of each enzyme were used in assays. After incubation, 50 µL of the reactions was used for quantification of reducing ends with the DNS assay.

To determine which products appeared after cleavage of SBA and DA, reactions were carried out by incubating the different enzymes with either SBA or DA or both substrates in 25 mM citrate–phosphate buffer at optimal temperature and pH for different time spans (0 h, 0.5 h, 1.5 h, 4 h, 8 h, 20 h). Thin-layer chromatography (TLC) was used for the determination of hydrolysis products as described in our previously study [52].

To explore the cleavage mode and preference towards side chains of arabinose-containing oligosaccharides, the enzymes were incubated with 0.25 g L^−1^ oligosaccharides, in reaction mixtures of 10 µL containing 25 mM citrate–phosphate buffer at their optimal temperature and pH for 12 h. The reactions were terminated by boiling at 100 °C for 10 min. The hydrolysis products were identified by high-performance anion-exchange chromatography with pulsed amperometric detection (HPAEC-PAD) as described by Liu et al*.* [52].

To reveal the substrate preference of each enzyme and synergistic effects of different enzymes, the single enzyme activities and combined activities of different enzymes were tested by using SBA and DA as substrates. The synergistic activities were tested by adding 75 nM endo-arabinanases or 150 nM exo-arabinofuranosidases to a 25 mM citrate–phosphate buffer (pH 5.5) containing 0.5 g L^−1^ SBA/DA followed by incubation at 45 °C for 24 h with shaking at 600 rpm. Reducing sugar released during the reaction was quantified by the DNS assay. The hydrolysis products and yields of arabinose obtained were quantified by using HPAEC-PAD referring to a standard curve obtained from a series of different arabinose concentrations (200 mg L^−1^, 100 mg L^−1^, 50 mg L^−1^, 25 mg L^−1^, 12.5 mg L^−1^).

## Supplementary Information

**Additional file 1:** **Table S1.** Bacterial community compositions of the biogas fermenter consortia revealed by 16S rRNA amplicon sequencing. **Table S2.** Raw data summary of shotgun sequencing and assemblage of positive fosmid clones.** Table S3.** Detailed information about all the putative CAZymes screened from the metagenomic libraries, including the position of ORFs on the corresponding contigs, the library origin, the KEGG annotation and blastnr identifier of each putative enzyme. **Table S4.** Information on heterologously expressed arabinosyl hydrolases. **Table S5.** Primers used for PCR amplification of putative arabinosyl hydrolase-encoding genes.** Figure S1.** Functional screening of recombinant clones of *E. coli *fosmid libraries using four different chromogenic substrates. Cleavage of the chromogenic substrates results in increased color intensity in the presence of recombinantly expressed activities, including carboxymethyl cellulase (CMCase, red color), xylanase (blue color), arabinoxylanase (blue color), arabinanase (blue color). **Figure S2.** Gene organization of representative gene clusters encoding fibrolytic enzymes targeting different plant polysaccharides. CAZyme genes were predicted by the use of the dbCAN and KEGG databases. **Figure S3.** Temperature, pH dependence and thermo-resistance of enzyme activities. Relative activities were calculated from DNS assay with SBA or DA as substrate or *p*NP assay with *p*NP-AF as substrate. The maximal activity was set as 100%. Standard reactions for searching temperature and pH optimum included 1 mM *p*NP-AF (143.69 nM MC57GH51 and 19.86 nM MC60GH51) or with 5 g L^-1^ DA (875.62 nM MC60GH43, 367.05 nM MC68GH43-2 and 465.25 nM MC72GH43-2) or SBA (MC68GH43-1), assays were carried out in 25 mM citrate phosphate buffer with pH between 4.0 and 9.0 and temperature between 25° C and 80°  C for 30 min, or with 1 mM *p*NP-AF for 10 min. For analysis of the enzymes’ thermo-resistance, the assays included 5 g L^-1^ of DA, 25 mM citrate phosphate buffer with 283.3 nM MC60GH43, 547.54 nM MC68GH43-2, 99.5 nM MC72GH43-2 or 5 g L^-1^ of SBA with 120.04 nM MC57GH51, 410.96 nM MC60GH51, 892.86 nM MC68GH43-1. The enzymes were incubated at their optimal temperature and pH for various time spans before measuring residual activity against SBA (MC57GH51, MC60GH51, MC68GH43-1) or DA (MC60GH43, MC68GH43-2, MC72GH43-2). The assays were performed in triplicates. **Figure S4.** Determination of kinetic parameters of arabinosyl hydrolases with SBA or DA as substrates. Standard reactions were performed by using three different concentrations of each enzyme (as indicated in figures) and various concentration of substrates (between 1 and 50 g L^-1^) at each enzyme’s optimal condition for different time periods according to requirement. (MC68GH43-1 and MC60GH43 for 2 h incubation, MC60GH51, MC68GH43-2, MC72GH43-2 for 40 min incubation, MC57GH51 for 1 h incubation). Error bars represent standard deviation of triplicates. *K*_m_ and *V*_max_ were calculated by using Microsoft Excel Solver, as described in material and methods.

## Data Availability

The datasets used and/or analyzed during the current study are available from the corresponding author on reasonable request.

## Abbreviations

CPH: Chromogenic polysaccharide hydrogel
ICB: Insoluble chromogenic biomass
CAZyme: Carbohydrate-active enzyme
ORFs: Open reading frames
GHs: Glycoside hydrolases
SBP: Sugar beet pulp
CBM: Carbohydrate-binding module
CGC: CAZyme gene clusters
*p*NP -AF: *p*-Nitrophenyl-α-l-arabinofuranoside
DA: Debranched arabinan
SBA: Sugar beet arabinan
AOS: Arabino-oligosaccharide
XOS: Xylo-oligosaccharide
AXOS: Arabinoxylo-oligosaccharide
HPAEC-PAD: High-performance anion-exchange chromatography with pulsed amperometric detection
TLC: Thin-layer chromatography
RGI: Rhamnogalacturonan I
