# Supplementary material for: Arabinan saccharification by biogas reactor metagenome-derived arabinosyl hydrolases
Source: Biotechnol Biofuels Bioprod. 2022 Nov 12;15:121. doi: 10.1186/s13068-022-02216-9 (PMC9655821; doi:10.1186/s13068-022-02216-9)
Supplement: Supplementary file 1 — Additional file 1: Table S1. Bacterial community compositions of the biogas fermenter consortia revealed by 16S rRNA amplicon sequencing. Table S2. Raw data summary of shotgun sequencing and assemblage of positive fosmid clones. Table S3. Detailed information about all the putative CAZymes screened from the metagenomic libraries, including the position of ORFs on the corresponding contigs, the library origin, the KEGG annotation and blastnr identifier of each putative enzyme. Table S4. Information on heterologously expressed arabinosyl hydrolases. Table S5. Primers used for PCR amplification of putative arabinosyl hydrolase-encoding genes. Figure S1. Functional screening of recombinant clones of E. coli fosmid libraries using four different chromogenic substrates. Cleavage of the chromogenic substrates results in increased color intensity in the presence of recombinantly expressed activities, including carboxymethyl cellulase (CMCase, red color), xylanase (blue color), arabinoxylanase (blue color), arabinanase (blue color). Figure S2. Gene organization of representative gene clusters encoding fibrolytic enzymes targeting different plant polysaccharides. CAZyme genes were predicted by the use of the dbCAN and KEGG databases. Figure S3. Temperature, pH dependence and thermo-resistance of enzyme activities. Relative activities were calculated from DNS assay with SBA or DA as substrate or pNP assay with pNP-AF as substrate. The maximal activity was set as 100%. Standard reactions for searching temperature and pH optimum included 1 mM pNP-AF (143.69 nM MC57GH51 and 19.86 nM MC60GH51) or with 5 g L-1 DA (875.62 nM MC60GH43, 367.05 nM MC68GH43-2 and 465.25 nM MC72GH43-2) or SBA (MC68GH43-1), assays were carried out in 25 mM citrate phosphate buffer with pH between 4.0 and 9.0 and temperature between 25° C and 80° C for 30 min, or with 1 mM pNP-AF for 10 min. For analysis of the enzymes’ thermo-resistance, the assays included 5 g L-1 of DA, 25 mM citrate phosphate buffer wi [file 13068_2022_2216_MOESM1_ESM.docx]

**Additional materials**

**Arabinan saccharification by biogas reactor metagenome-derived arabinosyl hydrolases**

Yajing Liu^1,2^ (yajing.liu@tum.de), Angel Angelov^1,3^(angelov@tum.de), Werner Feiler^1^(wernerfeiler@gmx.de), Melanie Baudrexl^1^(melanie.baudrexl@tum.de), Vladimir Zverlov^1#^(vladimir.zverlov@tum.de), Wolfgang Liebl^1,*^ (wliebl@tum.de), Sonja Vanderhaeghen^1^ (sonja_vanderhaeghen@web.de) ^*^

**Affiliation:**

^1^Chair of Microbiology, Technical University of Munich, Emil-Ramann-Straβe 4, D-85354 Freising-Weihenstephan, Germany

^2^Current address: Chair of Chemistry of Biogenic Resources, Technical University of Munich, Schulgasse 16, D-94315 Straubing, Germany.

^3^Current address: NGS Competence Center Tübingen, Universitätsklinikum Tübingen, Calwerstraße 7, D-72076 Tübingen, Germany

^4^Current address: IMGM laboratories, Lochhamer Straße 29a, 82152 Planegg, Germany.

^#^ Dr. Vladimir Zverlov passed away on 4^th^ March 2022

**^*^Corresponding authors**

Prof. Dr. Wolfgang Liebl

Chair of Microbiology, Technical University of Munich, Emil-Ramann-Straβe 4, D-85354 Freising-Weihenstephan, Germany

wliebl@tum.de

Dr. Sonja Vanderhaeghen

Chair of Microbiology, Technical University of Munich, Emil-Ramann-Straβe 4, D-85354 Freising-Weihenstephan, Germany

sonja_vanderhaeghen@web.de

**Table S1 Bacterial community compositions of the biogas fermenter consortia revealed by 16S rRNA amplicon sequencing.**

| **Phyla** | **MOD18 library** | | **T1T2 library** | |
| --- | --- | --- | --- | --- |
|  | **OUTs of V3 and V4** | **Percentage (%)** | **OUTs of V3 and V4** | **Percentage (%)** |
| Firmicutes | 16756 | 88.66 | 20185 | 82.12 |
| Thermotogae | 1187 | 6.28 | 4233 | 17.22 |
| Bacteroidetes | 938 | 4.96 | 8 | 0.03 |
| Proteobacteria | 0 | 0 | 1 | 0 |
| Others | 18 | 0.1 | 152 | 0.62 |
| Sum | 18899 | 1 | 24579 | 1 |

**Table S2** **Raw data summary of shotgun sequencing and assemblage of positive fosmid clones.**

| **Summary** | **All contigs after assemblage** | **Contigs with CAZy genes** | **All contigs after assemblage** | **Contigs with CAZy genes** |
| --- | --- | --- | --- | --- |
|  | **pCC1 fosmid** | **pCC1 fosmid** | **pCT3FK fosmid** | **pCT3FK fosmid** |
| No. of contigs (>=1000 bp) | 130 | 54 | 128 | 70 |
| Total length  (>=1000 bp) | 4750665 | 3968230 | 4805647 | 4164758 |
| Average length (bps) | 36543 | 73485 | 37544 | 59496 |
| GC content (%) | 50.04 | - | 49.91 | - |
| N50 | 92492 | - | 87225 | - |
| Longest contig (bps) | 327147 | 327147 | 327164 | 327164 |

**Table S3. Detailed information about all the putative CAZymes screened from the metagenomics library, including the position of ORFs on the corresponding contigs, the library origin, the KEGG annotation and blastnr identify of each putative enzyme.**

| **dbCAN** | **Name** | **Library of origin** | **KEGG** | **Blastnr identity** |
| --- | --- | --- | --- | --- |
| **GH43** |  |  |  |  |
| pCC1FOS_Contig41  (27753_30071) | xynD | SBP enriched fermenter microbial community | arabinoxylan arabinofuranohydrolase [EC:3.2.1.55] | 66.84% MULTISPECIES: family 43 glycosylhydrolase [unclassified *Fibrobacter*] |
| pCC1FOS_Contig41  (30291_32525) | xynD | SBP enriched fermenter microbial community | arabinoxylan arabinofuranohydrolase [EC:3.2.1.55] | 80.66% MULTISPECIES: carbohydrate-binding protein [unclassified *Fibrobacter*] |
| pCC1FOS_Contig60  (8873_9811) | lacZ | SBP enriched fermenter microbial community | beta-galactosidase [EC:3.2.1.23] | 64.69% family 43 glycosylhydrolase [*Gorillibacterium massiliense*] |
| pCC1FOS_Contig68  (3297_4850) | abnA | SBP enriched fermenter microbial community | arabinan endo-1,5-alpha-L-arabinosidase [EC:3.2.1.99] | 81.35% glycoside hydrolase family 43 protein [*Thermoclostridium stercorarium*] |
| pCC1FOS_Contig68  (4869_5849) | lacZ | SBP enriched fermenter microbial community | beta-galactosidase [EC:3.2.1.23] | 84.31% family 43 glycosylhydrolase [*Thermoclostridium stercorarium*] |
| pCC1FOS_Contig72  (1_1561) | abnA | SBP enriched fermenter microbial community | arabinan endo-1,5-alpha-L-arabinosidase [EC:3.2.1.99] | 65.27% hypothetical protein BK120_06600 [*Paenibacillus* sp. FSL A5-0031] |
| pCC1FOS_Contig72  (2960_5554) | abnA | SBP enriched fermenter microbial community | arabinan endo-1,5-alpha-L-arabinosidase [EC:3.2.1.99] | 75.21% S-layer domain protein [*Thermoanaerobacter mathranii* subsp. mathranii str. A3] |
| pCT3FK_Contig40  (25320_26750) | abnA | Thermophilic digestate, fermenter | arabinan endo-1,5-alpha-L-arabinosidase [EC:3.2.1.99] | 99.79% TPA: endo-alpha-(1->5)-L-arabinanase [*Firmicutes* bacterium] |
| pCT3FK_Contig40  (34791_35702) | abnA | Thermophilic digestate, fermenter | arabinan endo-1,5-alpha-L-arabinosidase [EC:3.2.1.99] | 80.47% TPA: endo-alpha-(1->5)-L-arabinanase [*Firmicutes* bacterium] |
| pCT3FK_Contig53  (19346_20782) | xynD | Thermophilic digestate, fermenter | arabinoxylan arabinofuranohydrolase [EC:3.2.1.55] | 83.86% family 43 glycosylhydrolase [*Thermoclostridium stercorarium]* |
| pCT3FK_Contig53  (20832_22727) | xynD | Thermophilic digestate, fermenter | arabinoxylan arabinofuranohydrolase [EC:3.2.1.55] | 61.72% Select seq ref\|WP_137182487.1\| family 43 glycosylhydrolase [*Paenibacillus* sp. SY21-1] |
| pCT3FK_Contig53  (22839_24615) | | Thermophilic digestate, fermenter | xylosidase | 64.18% Select seq gb\|ABD48561.1\| putative exo-xylanase [*Geobacillus thermoleovorans*] |
| pCT3FK_Contig68  (1489_3672) | xynD | Thermophilic digestate, fermenter | arabinoxylan arabinofuranohydrolase [EC:3.2.1.55] | 100.00% carbohydrate-binding protein [*Hungateiclostridium saccincola*] |
| pCT3FK_Contig68  (3698_5116) | xynD | Thermophilic digestate, fermenter | arabinoxylan arabinofuranohydrolase [EC:3.2.1.55] | 99.79% family 43 glycosylhydrolase [*Hungateiclostridium saccincola*] |
| pCT3FK_Contig83  (3191_5158) | | Thermophilic digestate, fermenter | arabinoxylan arabinofuranohydrolase [EC:3.2.1.55] | 100% family 43 glycosyl hydrolase [*Hungateiclostridium saccincola*] |
| **GH51** |  |  |  |  |
| pCC1FOS_Contig57  (20222_21700) | abfA | SBP enriched fermenter microbial community | alpha-L-arabinofuranosidase [EC:3.2.1.55] | 99.59% alpha-N-arabinofuranosidase [*Xylanivirga thermophila*] |
| pCC1FOS_Contig60  (10084_11595) | abfA | SBP enriched fermenter microbial community | alpha-L-arabinofuranosidase [EC:3.2.1.55] | 97.42%alpha-N-arabinofuranosidase [*Xylanivirga thermophila*] |
| **GH10** |  |  |  |  |
| pCC1FOS_Contig41  (32705_34759) | | SBP enriched fermenter microbial community | endo-1,4-beta-xylanase [EC:3.2.1.8] | 71.2% Select seq ref\|WP_088661344.1\| carbohydrate-binding protein [*Fibrobacter* sp. UWB2] |
| pCC1FOS_Contig41  (34983_36488) | | SBP enriched fermenter microbial community | endo-1,4-beta-xylanase [EC:3.2.1.8] | 73.72% carbohydrate-binding protein [*Fibrobacter* sp. UWR3] |
| pCC1FOS_Contig58  (2578_5235) | | SBP enriched fermenter microbial community | endo-1,4-beta-xylanase [EC:3.2.1.8] | 59.75% TPA: hypothetical protein [*Bacteroidales* bacterium] |
| pCC1FOS_Contig40  (20987_22144) | | Thermophilic digestate, fermenter | endo-1,4-beta-xylanase [EC:3.2.1.8] | 99.73% endo-1,4-xylanase [bacterium enrichment culture] |
| pCT3FK_Contig53  (6674_9940) | | Thermophilic digestate, fermenter | endo-1,4-beta-xylanase [EC:3.2.1.8] | 100% glycoside hydrolase [*Hungateiclostridium saccincola*] |
| pCT3FK_Contig55  (4567_6363) | | Thermophilic digestate, fermenter | endo-1,4-beta-xylanase [EC:3.2.1.8] | 99.83% carbohydrate-binding protein [*Hungateiclostridium saccincola*] |
| pCT3FK_Contig57  (16610_17836) | | Thermophilic digestate, fermenter | endo-1,4-beta-xylanase [EC:3.2.1.8] | 66.92% 1,4-beta-xylanase [*Halanaerobium saccharolyticum*] |
| pCT3FK_Contig58  (1_1195) | | Thermophilic digestate, fermenter | endo-1,4-beta-xylanase [EC:3.2.1.8] | 92.87% glycoside hydrolase family 11 protein [*Hungateiclostridium saccincola*] |
| pCT3FK_Contig61  (10525_11592) | | Thermophilic digestate, fermenter | endo-1,4-beta-xylanase [EC:3.2.1.8] | 58.43% endo-1,4-beta-xylanase [*Paenibacillus nanensis*] |
| pCT3FK_Contig61  (8034_9023) | | Thermophilic digestate, fermenter | endo-1,4-beta-xylanase [EC:3.2.1.8] | 59.08% endo-1,4-beta-xylanase [*Paenibacillus bovis*] |
| pCT3FK_Contig61  (9250_10428) | | Thermophilic digestate, fermenter | endo-1,4-beta-xylanase [EC:3.2.1.8] | 46.15% Select seq ref\|WP_088078005.1\| endo-1,4-beta-xylanase [*Bacillus alkalitelluris*] |
| pCT3FK_Contig82  (1700_4123) | | Elephant feces | endo-1,4-beta-xylanase [EC:3.2.1.8] | 100.00% carbohydrate-binding protein [*Hungateiclostridium saccincola*] |
| pCT3FK_Contig94  (1095_1781) | | Elephant feces | endo-1,4-beta-xylanase [EC:3.2.1.8] | 70.93% carbohydrate-binding protein [*Gracilibacillus thailandensis*] |
| **GH2** |  |  |  |  |
| pCC1FOS_Contig56  (21841_22494) | | SBP enriched fermenter microbial community | beta-mannosidase [EC:3.2.1.25] | 100%Select seq ref\|WP_144403490.1\| glycoside hydrolase family 2 protein [*Defluviitoga tunisiensis*] |
| pCC1FOS_Contig94  (1_1849) | | SBP enriched fermenter microbial community | beta-mannosidase [EC:3.2.1.25] | 99.35% glycoside hydrolase family 2 protein [*Defluviitoga tunisiensis*] |
| pCT3FK_Contig33  (32489_35470) | lacZ | - | beta-galactosidase [EC:3.2.1.23] | 100% TPA: beta-galactosidase [*Shigella* sp.] |
| pCT3FK_Contig102  (1_632) | | - | beta-mannosidase [EC:3.2.1.25] | 99.52% glycoside hydrolase family 2 protein [*Defluviitoga tunisiensis*] |
| pCT3FK_Contig128  (1_1012) | | - | beta-mannosidase [EC:3.2.1.25] | 99.70% glycoside hydrolase family 2 protein [*Defluviitoga tunisiensis*] |
| **GH11** |  |  |  |  |
| pCC1FOS_Contig45  (18784_20319) | | SBP enriched fermenter microbial community | endo-1,4-beta-xylanase [EC:3.2.1.8] | 99.22% Select seq sp\|P33558.2\| RecName: Full=Endo-1,4-beta-xylanase A; Short=Xylanase A; AltName: Full=1,4-beta-D-xylan xylanohydrolase A; Flags: Precursor [*Thermoclostridium stercorarium*] |
| pCT3FK_Contig58  (1_1195) | | Thermophilic digestate, fermenter | endo-1,4-beta-xylanase [EC:3.2.1.8] | 92.87% glycoside hydrolase family 11 protein [*Hungateiclostridium saccincola*] |
| pCT3FK_Contig82  (1700_4123) | | Elephant feces | endo-1,4-beta-xylanase [EC:3.2.1.8] | 100.00% carbohydrate-binding protein [*Hungateiclostridium saccincola*] |
| pCT3FK_Contig94  (1095_1781) | | Elephant feces | endo-1,4-beta-xylanase [EC:3.2.1.8] | 70.93%carbohydrate-binding protein [*Gracilibacillus thailandensis*] |
| **GH3** |  |  |  |  |
| pCC1FOS_Contig93  (754_2180) | bglX | SBP enriched fermenter microbial community | beta-glucosidase [EC:3.2.1.21] | 98.53% Select seq ref\|WP_144403489.1\| glycosyl hydrolase [*Defluviitoga tunisiensis*] |
| pCT3FK_Contig40  (13964_16135) | bglX | Thermophilic digestate, fermenter | beta-glucosidase [EC:3.2.1.21] | 99.86% Select seq gb\|AJO67863.1\| beta-xylosidase [bacterium enrichment culture] |
| pCT3FK_Contig102  (635_2230) | bglX | Elephant feces | beta-glucosidase [EC:3.2.1.21] | 98.87% glycosyl hydrolase [*Defluviitoga tunisiensis*] |
| **GH9** |  |  |  |  |
| pCT3FK_Contig63  (677_2287) | | Thermophilic digestate, fermenter | endoglucanase [EC:3.2.1.4] | 100.00% Select seq gb\|AXR85444.1\| glycoside hydrolase family 9 endoglucanase [uncultured bacterium] |
| pCT3FK_Contig70  (1716_3551) | | Thermophilic digestate, fermenter | endoglucanase [EC:3.2.1.4] | 100% glycoside hydrolase [*Hungateiclostridium saccincola*] |
| pCT3FK_Contig75  (820_4731) | | Thermophilic digestate, fermenter | endoglucanase [EC:3.2.1.4] | 99.77% hypothetical protein [*Hungateiclostridium saccincola*] |
| **GH13** |  |  |  |  |
| pCT3FK_Contig83  (831_2270) | |  | starch synthase (maltosyl-transferring) [EC:2.4.99.16](Second best) | 100%maltodextrin glycosyltransferase [*Defluviitoga tunisiensis*] |
| pCT3FK_Contig111  (1_570) | |  | starch synthase (maltosyl-transferring) [EC:2.4.99.16](Second best) | 99.47% maltodextrin glycosyltransferase [*Defluviitoga tunisiensis*] |
| **GH4** |  |  |  |  |
| pCC1FOS__Contig56  (1592_3049) | melA | SBP enriched fermenter microbial community | alpha-galactosidase [EC:3.2.1.22] | 100.00% Select seq ref\|WP_045087944.1\| alpha-glucosidase/alpha-galactosidase [*Defluviitoga tunisiensis*] |
| **GH106** |  |  |  |  |
| pCC1FOS__Contig56  (4383_7496) | | SBP enriched fermenter microbial community |  | 99.23% hypothetical protein [*Defluviitoga tunisiensis*] |
| **GH73** |  |  |  |  |
| pCC1FOS__Contig57  (518_1327) | amiABC | SBP enriched fermenter microbial community | N-acetylmuramoyl-L-alanine amidase [EC:3.5.1.28] (second best) | 100.00% glucosaminidase domain-containing protein [*Xylanivirga thermophila*] |
| **GH6** |  |  |  |  |
| pCC1FOS__Contig66  (2282_4735) | clpC | SBP enriched fermenter microbial community | ATP-dependent Clp protease ATP-binding subunit ClpC | 100% ATP-dependent Clp protease ATP-binding subunit [*Xylanivirga thermophila*] |
| **GH127** |  |  |  |  |
| pCC1FOS__Contig72  (5640_7598) | | SBP enriched fermenter microbial community | uncharacterized protein | 64.89% glycoside hydrolase family 127 protein [*Petroclostridium xylanilyticum*] |
| pCC1FOS__Contig72  (7624_7782) | | SBP enriched fermenter microbial community |  | 78.00% Select seq gb\|PYE64535.1\| hypothetical protein C7489_12345 [*Paenibacillus* sp. OV191] |
| pCC1FOS__Contig72  (7745_8221) | | SBP enriched fermenter microbial community | alpha-galactosidase [EC:3.2.1.22] | 62.33% glycoside hydrolase family 27 protein [*Paenibacillus prosopidis*] |
| **GH94** |  |  |  |  |
| pCC1FOS__Contig83  (2629_3328) | | SBP enriched fermenter microbial community | cellobiose phosphorylase [EC:2.4.1.20] | 99.57% glycosyl transferase [*Defluviitoga tunisiensis*] |
| **GH1** |  |  |  |  |
| pCC1FOS_Contig93  (36_536) | bglB | SBP enriched fermenter microbial community | beta-glucosidase [EC:3.2.1.21] | 99.4% beta-glucosidase [*Defluviitoga tunisiensis*] |
| **GH24** |  |  |  |  |
| pCT3FK_Contig33  (18118_18612) | | - | lysozyme [EC:3.2.1.17] | 100.00% Select seq ref\|WP_001070143.1\| MULTISPECIES: lysozyme [Bacteria] |
| **GH36** |  |  |  |  |
| pCT3FK_Contig33  (31184_32437) | lacY | - | MFS transporter, OHS family, lactose permease | 100.00% Select seq ref\|WP_000291549.1\| MULTISPECIES: lactose permease [*Enterobacteriaceae*] |
| **GH67** |  |  |  |  |
| pCT3FK_Contig40  (11877_13895) | aguA | Thermophilic digestate, fermenter | alpha-glucuronidase [EC:3.2.1.139] | 99.55% TPA: alpha-glucuronidase [*Firmicutes bacterium*] |
| **CE1** |  |  |  |  |
| pCT3FK_Contig53  (6674_9940) | | Thermophilic digestate, fermenter | endo-1,4-beta-xylanase [EC:3.2.1.8] | 100%glycoside hydrolase [*Hungateiclostridium saccincola*] |
| pCT3FK_Contig68  (646_1449) | | Thermophilic digestate, fermenter |  | 100.00% esterase family protein [*Hungateiclostridium saccincola*] |
| pCT3FK_Contig82  (1_1429) | xynA | Elephant feces | endo-1,4-beta-xylanase [EC:3.2.1.8] (second best) | 100.00% carbohydrate-binding protein [*Hungateiclostridium saccincola*] |
| **CE4** |  |  |  |  |
| pCT3FK_Contig58  (1_1195) | | Thermophilic digestate, fermenter | endo-1,4-beta-xylanase [EC:3.2.1.8] | 92.87% glycoside hydrolase family 11 protein [*Hungateiclostridium saccincola*] |
| pCT3FK_Contig82  (1700_4123) | | Elephant feces | endo-1,4-beta-xylanase [EC:3.2.1.8] | 100.00% carbohydrate-binding protein [*Hungateiclostridium saccincola*] |
| pCT3FK_Contig94  (1095_1781) | | Elephant feces | endo-1,4-beta-xylanase [EC:3.2.1.8] | 70.93% Select seq ref\|WP_153836669.1\| carbohydrate-binding protein [*Gracilibacillus thailandensis*] |
| **GH16** |  |  |  |  |
| pCT3FK_Contig68  (5429_6430) | exoK | Thermophilic digestate, fermenter | endo-1,3-1,4-beta-glycanase ExoK [EC:3.2.1.-] | 100%Select seq ref\|WP_101301458.1\| family 16 glycosylhydrolase [*Hungateiclostridium saccincola*] |
| **GH30** |  |  |  |  |
| pCT3FK_Contig68  (7788_9389) | | Thermophilic digestate, fermenter |  | 99.81% hypothetical protein [*Hungateiclostridium saccincola*] |
| **GH8** |  |  |  |  |
| pCT3FK_Contig70  (114_1295) | bcsZ | Thermophilic digestate, fermenter | endoglucanase [EC:3.2.1.4] | 99.74% hypothetical protein [*Hungateiclostridium saccincola*] |
| **GH18** |  |  |  |  |
| pCT3FK_Contig70  (3623_5536) | | Thermophilic digestate, fermenter | yaaH；spore germination protein(second best) | 100.00% Select seq ref\|WP_105367824.1\| LysM peptidoglycan-binding domain-containing protein [*Hungateiclostridium saccincola*] |
| **CE6** |  |  |  |  |
| pCT3FK_Contig70  (7432_8478) | | Thermophilic digestate, fermenter | pnbA；para-nitrobenzyl esterase [EC:3.1.1.-] (second best) | 99.71% Select seq ref\|WP_101302863.1\| sialate O-acetylesterase [*Hungateiclostridium saccincola*] |
| **CE11** |  |  |  |  |
| pCT3FK_Contig81  (1_297) |  | - | fabZ; 3-hydroxyacyl-[acyl-carrier-protein] dehydratase [EC:4.2.1.59] | 100.00% 3-hydroxyacyl-ACP dehydratase FabZ [*Hungateiclostridium saccincola*] |

*The values in brackets represent the position of the gene on the contig.

**Table S4** **Information on heterologously expressed arabinosyl hydrolases.**

| **Name** | **KEGG annotation** | **Gene Size (bps)** | **MW (kDa)** | **pI** | **SignalP** |  |
| --- | --- | --- | --- | --- | --- | --- |
|  |  |  |  |  |  |  |
| MC57GH51 | α-l-arabinofuranosidase  [EC: 3.2.1.55] | 1500 | 56.65 | 5.56 | N |  |
|  |  |  |  |  |  |  |
| MC60GH51 | Arabinan endo-1,5-α-l-arabinosidase  [EC: 3.2.1.99] | 1533 | 58.4 | 5.10 | N |  |
| MC68GH43-1 | Arabinan endo-1,5-α-l-arabinosidase  [EC: 3.2.1.99] | 1575 | 58.8 | 5.06 | N |  |
| MC60GH43 | α-L-arabinofuranosidase  [EC: 3.2.1.55] | 960 | 36.71 | 5.21 | N |  |
|  |  |  |  |  |  |  |
| MC68GH43-2 | Arabinan endo-1,5-α-l-arabinosidase  [EC: 3.2.1.99] | 1002 | 39.23 | 6.29 | N |  |
| MC72GH43-1 | Arabinan endo-1,5-α-l-arabinosidase  [EC: 3.2.1.99] | 1407 | 52.18 | 4.36 | N |  |
| MC72GH43-2 | Arabinan endo-1,5-α-l-arabinosidase  [EC: 3.2.1.99] | 2580 | 95.88 | 4.51 | N |  |

Note: Molecular weight and isoelectric point (pI) of each protein were predicted by clone manager software; Signal peptide (SignalP) was predicted by SignalP-5.0; Glycoside hydrolase family and activity of each enzyme was predicted by use of the dbCAN and KEGG database, respectively.

**Table S5 Primers used for PCR amplification of putative arabinosyl hydrolase-encoding genes.**

| **Name of Genes** | **Forward Primer** | **Reverse Primer** |
| --- | --- | --- |
| MC57GH51 | CTTTAAGAAGGAGATATACAATGATAAATGTAACTATCAATGCAG | TCAGTGGTGGTGGTGGTGGTGCGCGCAAACCTCTAAAACAAC |
| MC60GH51 | CTTTAAGAAGGAGATATACAATGTCAAATACTCAAAAAGCAATGATG | TCAGTGGTGGTGGTGGTGGTGCGCCTTCGCTAGCCTTATAACATTCC |
| MC68GH43-1 | CTTTAAGAAGGAGATATACAATGAGGAAAAAAGTCATTTGGCTGG | TCAGTGGTGGTGGTGGTGGTGCGCTTCAATAGCCCAAATACCGC |
| MC60GH43 | CTTTAAGAAGGAGATATACAATGAGCAAATCAGTAAATTTTG | TCAGTGGTGGTGGTGGTGGTGCGCATATGTAAATACAGGGTTACCAG |
| MC68GH43-2 | CTTTAAGAAGGAGATATACAATGATGGAACGAAACTTGGTAAAAAC | TCAGTGGTGGTGGTGGTGGTGCGCACCATATACCTGTTTCCGG |
| MC72GH43-1 | CTTTAAGAAGGAGATATACAATGAATTGGGAGTTAATAGCGTCAG | TCAGTGGTGGTGGTGGTGGTGCGCTTCTATAGGTAGGCCTAAAG |
| MC72GH43-2 | CTTTAAGAAGGAGATATACAATGTTGACAATTACATTTGTATC | TCAGTGGTGGTGGTGGTGGTGCGCCACTTCAGGAATTTCACCTG |
| Standard pCC1 Fosmid Primers | GGATGTGCTGCAAGGCGATTAAGTTGG | CTCGTATGTTGTGTGGAATTGTGAGC |
| Standard pCT3FK Fosmid Primers | GTAATACGACTCACTATAGGGCG | GTAAAACGACGGCCAGTGCCAAGC |


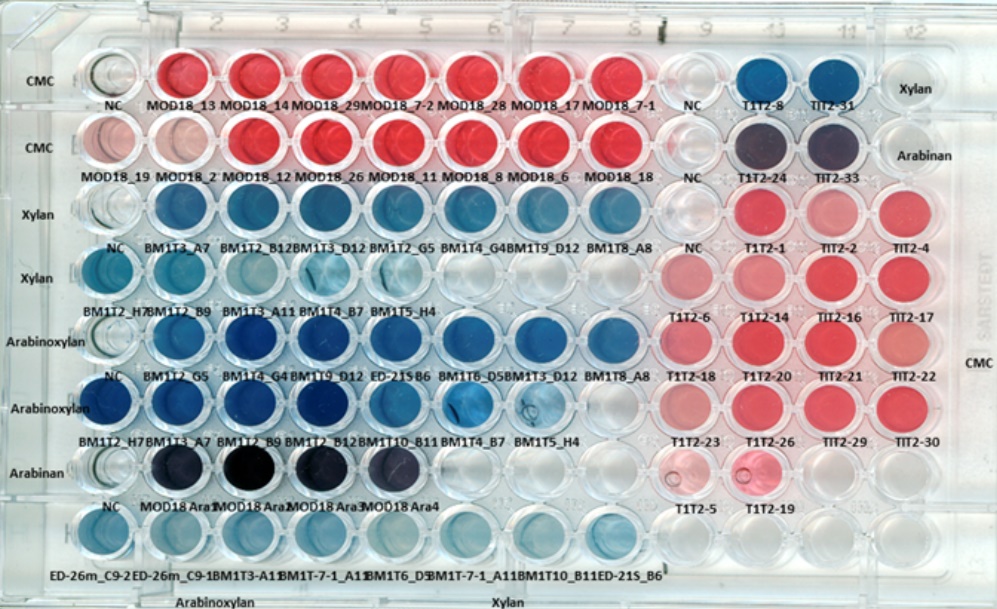


**Fig. S1. Functional screening of recombinant clones of *E. coli* fosmid libraries using four different chromogenic substrates.** Cleavage of the chromogenic substrates results in increased color intensity in the presence of recombinantly expressed activities, including carboxymethyl cellulase (CMCase, red color), xylanase (blue color), arabinoxylanase (blue color), arabinanase (blue color).


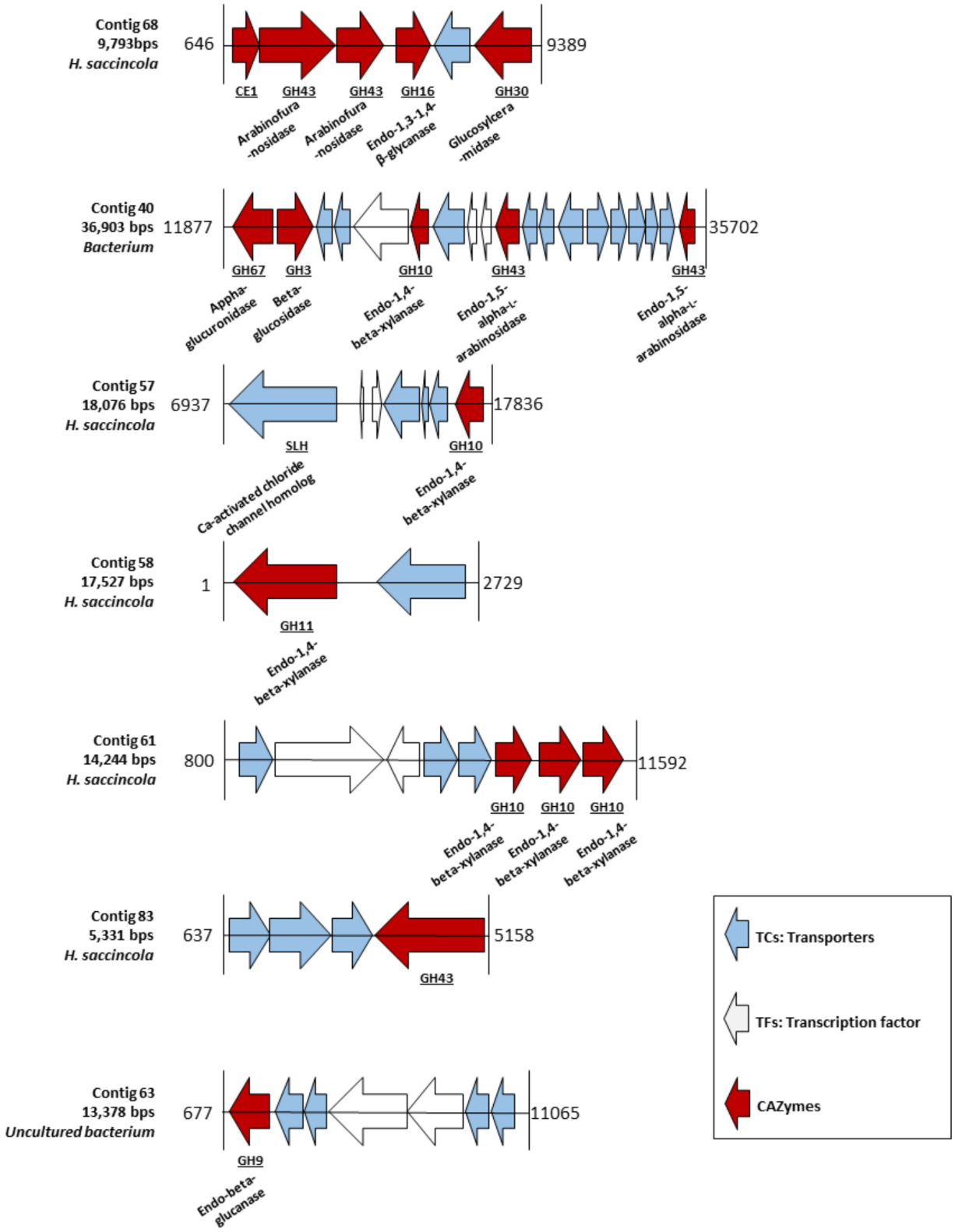


**Fig. S2. Gene organization of representative gene clusters encoding fibrolytic enzymes targeting different plant polysaccharides.** CAZyme genes were predicted by the use of the dbCAN and KEGG databases.


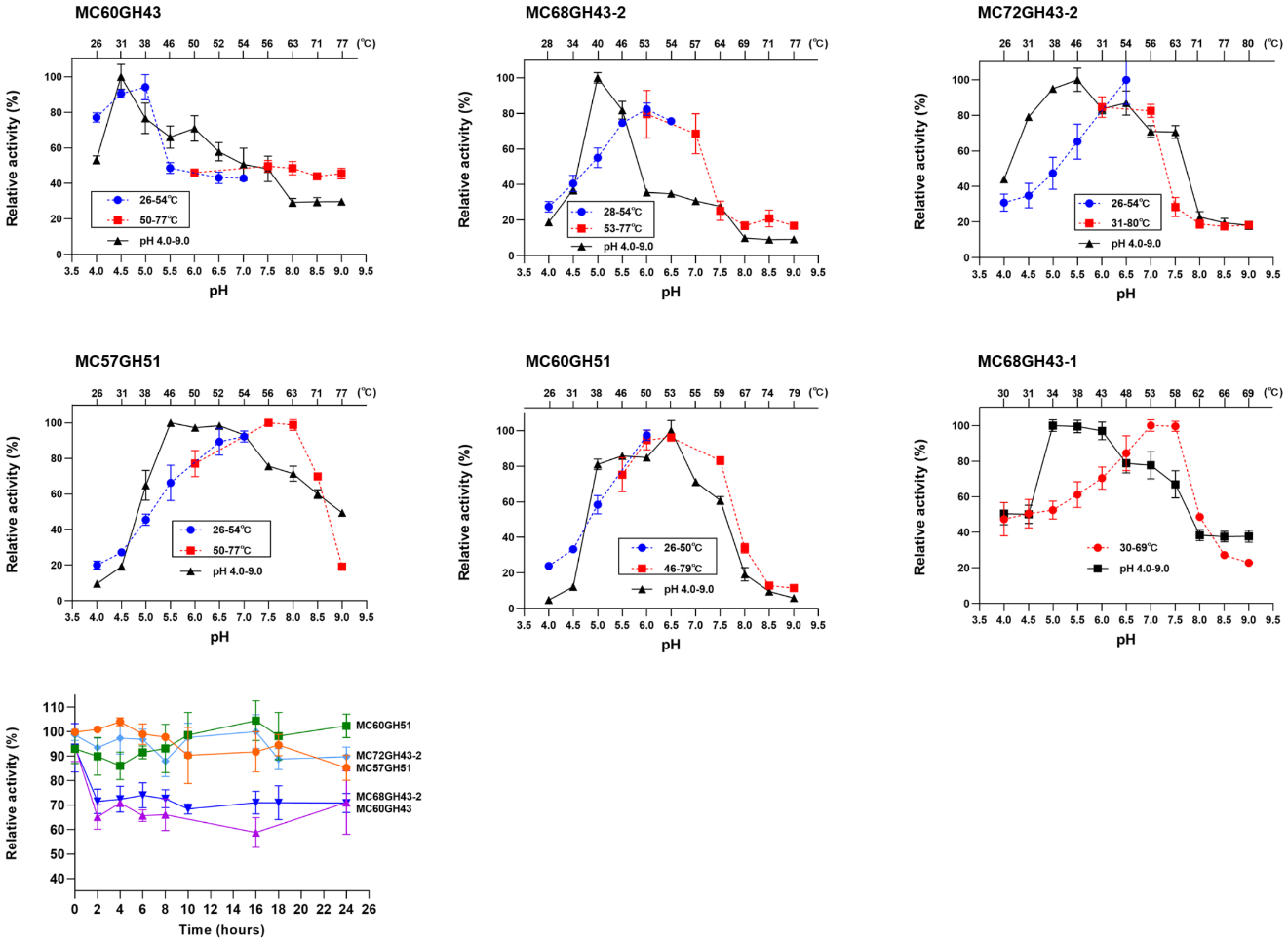


(C)

(F)

(E)

(G)

(B)

(D)

(A)

**Fig. S3. Temperature, pH dependence and thermo-resistance of enzyme activities.** Relative activities were calculated from DNS assay with SBA or DA as substrate or *p*NP assay with *p*NP-AF as substrate. The maximal activity was set as 100%. Standard reactions for searching temperature and pH optimum included 1 mM *p*NP-AF (143.69 nM MC57GH51 and 19.86 nM MC60GH51) or with 5 g L^-1^ DA (875.62 nM MC60GH43, 367.05 nM MC68GH43-2 and 465.25 nM MC72GH43-2) or SBA (MC68GH43-1), assays were carried out in 25 mM citrate phosphate buffer with pH between 4.0 and 9.0 and temperature between 25 ℃ to 80 ℃ for 30 min, or with 1 mM *p*NP-AF for 10 min. For searching enzymatic thermo-resistance, the assays included 5 g L^-1^ of DA, 25 mM citrate phosphate buffer with 283.3 nM MC60GH43, 547.54 nM MC68GH43-2, 99.5 nM MC72GH43-2 or 5 g L^-1^ of the SBA with 120.04 nM MC57GH51, 410.96 nM MC60GH51, 892.86 nM MC68GH43-1. The assays were performed in triplicates.

(B)

(A)


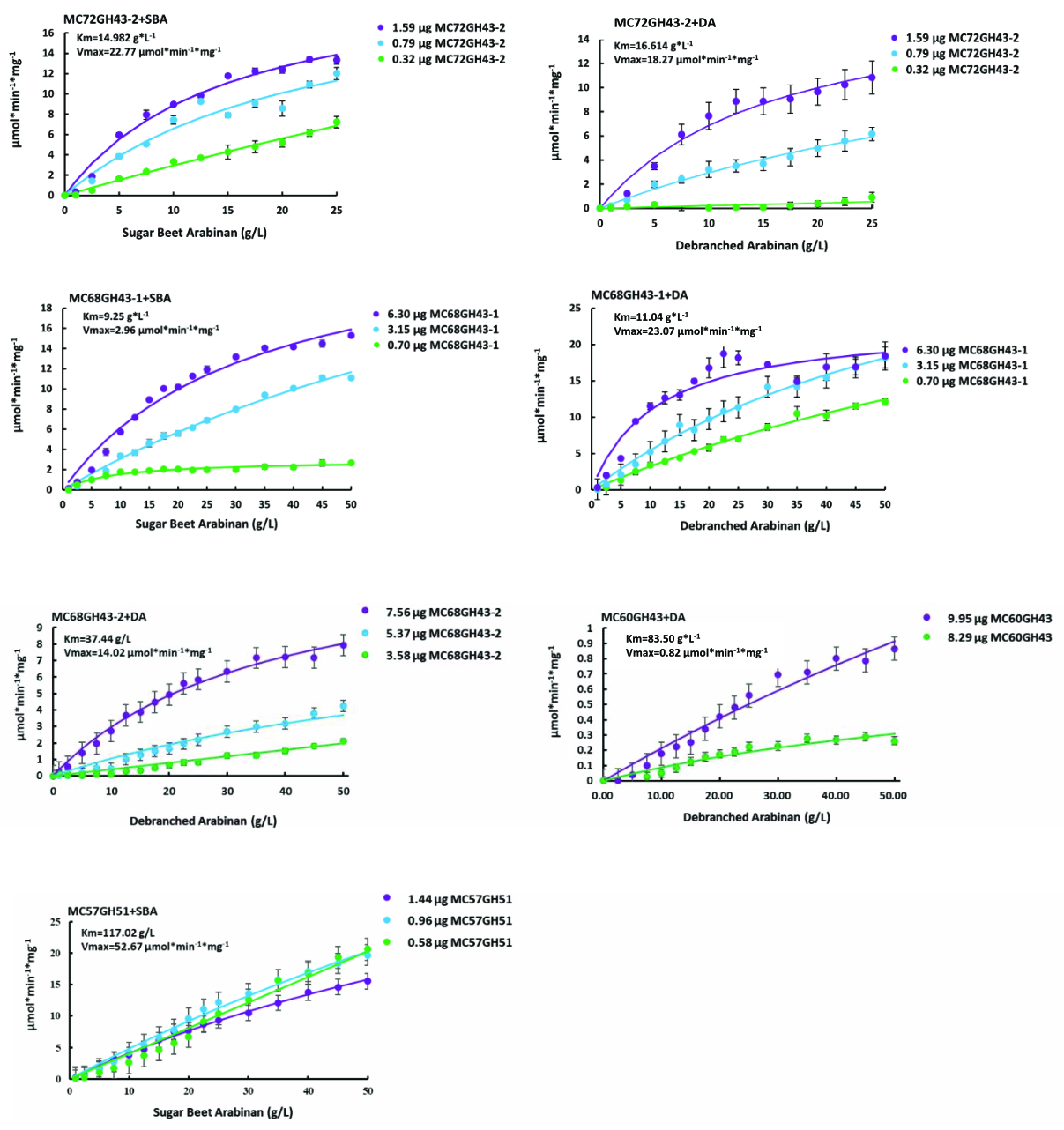


(G)

(F)

(E)

(D)

(C)

**Fig. S4. Determination of kinetic parameters of arabinosyl hydrolases with SBA or DA as substrates.** Standard reactions were performed by using three different concentrations of each enzyme (as indicated in figures) and various concentration of substrates (between 1 to 50 g L^-1^) at each enzyme’s optimal condition for different time periods according to requirement. (MC68GH43-1 and MC60GH43 for 2 h incubation, MC60GH51, MC68GH43-2, MC72GH43-2 ^^for 40 min incubation, MC57GH51 for 1 h incubation). Error bars represent standard deviation of triplicates. K_m_ and V_max_ were calculated by using Microsoft Excel Solver, as described in material and methods.
